# GOLM1 is related to the inflammatory/immune nature of uveal melanoma and acts as a promising indicator for prognosis and immunotherapy response

**DOI:** 10.3389/fgene.2022.1051168

**Published:** 2022-11-18

**Authors:** Xin Liang, Yu Yin, Ning Li

**Affiliations:** ^1^ Department of Ophthalmology, Shanghai General Hospital, Shanghai, China; ^2^ Department of Pathology, Anhui Medical University, Hefei, China; ^3^ Department of Ophthalmology, Anhui Medical University, Hefei, China

**Keywords:** GOLM1, inflammation, tumor microenvironment, immunotherapy, prognosis

## Abstract

**Purpose:** Inflammatory/immune-related features are associated with the immunotherapy and prognosis of uveal melanoma (UVM). In this study, we systematically analyzed the correlation between GOLM1 and the inflammatory/immune nature of UVM and explored its potential value in predicting prognosis and guiding immunotherapy for UVM patients.

**Methods:** A total of 143 UVM patients were enrolled in the current study. The differentially expressed genes between the GOLM1-low expression (LEXP) and GOLM1-high expression (HEXP) subgroups were calculated by the “limma” package and further annotated to reveal the key pathways by the “ClusterProfiler” package. Immunocyte infiltration was evaluated by single-sample gene set enrichment analysis, while the potential response to immunotherapy was realized by subclass mapping analysis. Moreover, tumor tissue sections from 23 UVM patients were collected and stained for GOLM1 (1:300; cat# DF8100, Affinity Biosciences), PD-L1 (1:250; cat# ab213524, Abcam), PD-1 (1:100; cat# ab52587, Abcam), CTLA-4 (1:300; cat# DF6793, Affinity Biosciences), and IFN-γ (1:300; cat# DF6045, Affinity Biosciences).

**Results:** We found that higher expression of GOLM1 correlated with an unfavorable prognosis in UVM patients. Multivariate Cox regression analysis suggested that GOLM1 served as a prognostic factor independent of clinicopathological parameters. Notably, we found that the expression of PD-1, PD-L1, IFN-γ, and CTLA4 was higher in the GOLM1-high subgroup than in the GOLM1-low expression subgroup at the mRNA level and was subsequently validated at the protein level by immunohistochemistry. Gene pattern and SubMap analyses confirmed the indicator role of GOLM1 in predicting immunotherapy response in UVM.

**Conclusion:** Taken together, GOLM1 is a novel prognostic marker, and it can be employed to predict the overall survival outcomes and treatment responses of anti-PD-1/PD-L1 and anti-CTLA4 therapies for UVM patients.

## Background

It has been reported that immune infiltrates are detectable in the majority of primary and metastatic uveal melanoma (UVM) (Krishna et al., 2017; Qin et al., 2020). The progression and metastasis of UVM are associated with inflammatory infiltration. Some UVMs with genetic aberrations, such as loss of one copy of chromosome 3 and BAP1 protein, produce inflammatory mediators. CD8^+^, CD4^+^, Foxp3^+^ T cells and macrophages are recruited and activated, resulting in the generation of more inflammatory mediators and a cancer-related tumor-promoting inflammatory microenvironment (Bronkhorst and Jager, 2012). The activation of nuclear factor-κB (NF-κB) was influenced, and additional proinflammatory multifunctional chemokines and cytokines were released, which affected infiltrating T lymphocytes (TILs) and tumor-associated macrophages (TAMs) in UVM. These driving T lymphocyte and macrophage infiltration are tightly linked to tumor recurrence and survival (Gezgin et al., 2017).

GOLM1 is a Golgi membrane protein mainly expressed in epithelial cells and upregulated in response to viral infection, with a coding gene of 3,042 bp in full length (Kladney et al., 2000). As reported, GOLM1 can be expressed in most human tissues, mainly in epithelial cells. GOLM1 is synthesized in the endoplasmic reticulum and is primarily transported to the cis-Golgi and is also a secreted protein that is detected in blood and urine. Until now, the biological function of GOLM1 in cells and tissues has not been entirely clear. Some studies have suggested that GOLM1 may play an important role in supporting normal cell function. In addition, the critical implication of GOLM1 in the prevention, prediction, and treatment of cancer has been established (Sun et al., 2011). Additionally, increased expression of GOLM1 is associated with some malignant biological characteristics and is an independent prognostic factor for poor overall survival (Sun et al., 2011; Bao et al., 2013; Chen et al., 2013). Notably, Kim et al. (2012) found that GOLM1 has a potential impact on inhibiting IL-12 production by dendritic cells, thus promoting tumor progression by preventing T cell responses in hepatocellular carcinoma. Consistently, Zhang et al. (2017a) revealed that host innate immune processes could be repressed by GOLM1, which will enhance the replication of the hepatitis C virus (HCV), thus driving the pathogenesis of hepatocellular carcinoma. Furthermore, increasing evidence suggests that GOLM1 regulates the STAT3 pathway, which is upstream of PD-L1 (Wölfle et al., 2011; Song et al., 2018; Zhang et al., 2019; Yan et al., 2020). Thus, it makes sense to regulate PD-L1 expression through targeting GOLM1. These findings highlight the potential role of GOLM1 in regulating immune responses in cancers.

However, the connection between GOLM1 and UVM, particularly its role in regulating inflammatory/immune responses in this tumor, has not been investigated. Herein, a higher expression level of GOLM1 was found in UVM patients than in controls. GOLM1 was considered an independent prognostic factor after the adjustment of clinicopathological features. In addition, the highly co-expressed genes of GOLM1 were primarily enriched in inflammatory/immune-related pathways, and patients with higher expression of GOLM1 might be more sensitive to anti-CTLA4 and anti-PD-1/PD-L1 immunotherapies. Our results provide a novel marker for clinical prediction, prognosis, and immunotherapy guidance for UVM patients.

## Materials and methods

### Patient summary

In this study, a total of 193 UVM patients were enrolled for analysis, from four cohorts, TCGA-UVM (*n* = 80), GSE21138 (*n* = 63), GSE84976 (*n* = 27) and a real-world cohort (*n* = 23), the basic information of these four cohorts listed in Table 1. For TCGA-UVM, the GDC platform was used to download the gene expression profile and clinical information *via* the “TCGAbiolinks” package; the GENCODE27 annotation file realized the further annotation of mRNA gene symbols. For GSE21138 and GSE84976, the gene expression profile and clinical features of whom came from the Gene Expression Omnibus platform (http://www.ncbi.nlm.nih.gov/geo/). Furthermore, the expression of GOLM1 in pan-cancer, the comparison of GOLM1 in normal and tumor tissues, and the GOLM1 level to overall survival (OS) for each cancer type were explored *via* the online website (http://sangerbox.com/).

**TABLE 1:** Basic informal of enrolled cohorts.

	TCGA-UVM (N = 80)	GSE21138 (N = 63)	GSE84976 (N = 27)	Real-world (N = 23)	Method	*p*-value
Age, months					Kruskal–Wallis	0.929
Mean (SD)	61.7 (13.9)	61.0 (12.3)	62.2 (15.2)	61.3 (13.0)		
Median [Min, Max]	61.5 [22.0, 86.0]	62.1 [28.6, 85.0]	68.0 [28.0, 84.0]	60.0 [39.0, 85.0]		
Gender					Chi-square	0.238
Female	35 (43.8%)	24 (38.1%)	—	15 (57.7%)		
Male	45 (56.3%)	39 (61.9%)	—	11 (42.3%)		
Basal diameter, mm					Kruskal–Wallis	0.044
Mean (SD)	16.9 (3.47)	15.4 (3.78)	—	15.9 (8.88)		
Median [Min, Max]	17.0 [7.79, 25.0]	15.0 [9.00, 23.0]	—	15.0 [4.00, 35.0]		
Missing	1 (1.3%)	10 (15.9%)	—	3 (11.5%)		
Thickness, mm					Kruskal–Wallis	0.036
Mean (SD)	10.4 (2.81)	11.7 (2.02)	—	13.3 (7.96)		
Median [Min, Max]	10.5 [4.00, 16.0]	11.7 [6.00, 17.0]	—	12.0 [3.00, 32.0]		
Missing	0 (0%)	0 (0%)	—	3 (11.5%)		
Extrascleral extension					Chi-square	1.000
Unknown	5 (6.3%)	10 (15.9%)	—	—		
No	68 (85.0%)	48 (76.2%)	—	—		
Yes	7 (8.8%)	5 (7.9%)	—	—		

### Signaling pathway annotation

For GOLM1-low (LEXP) and GOLM1-high (HEXP) subgroups, the “limma” package was used to identify differentially expressed genes (DEGs) between them, accompanied by further filtering with the requirement of fold-change >1 and *p*-value <0.01. Kyoto Encyclopedia of Genes and Genomes (KEGG), Gene Ontology (GO), and HALLMARK background gene set signatures were downloaded from MSigDB (Subramanian et al., 2005). We further implemented DEG enrichment annotation *via* the “ClusterProfiler” package (Yu et al., 2012).

### Tumor immunocyte infiltration evaluation

To reveal the immunocyte infiltration status among UVM patients, we collected 28 immunocyte signatures from a previous study (Yoshihara et al., 2013). With the support of the “GSVA” R package, a single sample gene set enrichment analysis (ssGSEA) (Barbie et al., 2009; Hanzelmann et al., 2013) was conducted. Then, the normalized enrichment score (NES) of 28 immunocyte signatures was calculated for each UVM sample.

### Predicting the response to immunotherapy

For an examination of individuals’ possibility of responding to immunotherapy, the expression value of 795 response-specific genes was generated from a melanoma cohort in which anti-PD-1 or anti-CTLA-4 checkpoint therapy was applied (Chen et al., 2016). The comparison between GOLM1 groups and immunotherapy groups was realized by subclass mapping analysis, thereby identifying potential responders in GOLM1 groups (Hoshida et al., 2007).

### Immunohistochemistry

We collected basic information and paraffin tissue sections of 23 patients with ocular melanoma from the First and the Second Affiliated Hospital of Anhui Medical University (Supplementary Table S1). The Institutional Review Board of Anhui Medical University approved this study. Paraffin tissues were cut into five-µm-thick sections for histological analysis. Xylene and different concentrations of alcohol were used for deparaffinization and dehydration of the sections. Then, the sections were placed in boiling antigen retrieval solution for 15 min to complete antigen retrieval of those tissues. After the sections were equilibrated to room temperature in antigen retrieval solution, 3% hydrogen peroxide solution was used for catalase blocking, and sections were placed into 0.1% potassium permanganate solution at 37°C for 40 min, followed by placement into 1% oxalic acid solution for decolorization. After washing 3 times with the washing solution, sections were incubated with primary antibodies [PD-L1 (1:250; cat^#^ab213524, Abcam), PD-1 (1:100; cat^#^ab52587, Abcam), CTLA-4 (1:300; cat^#^DF6793, Affinity Biosciences), IFN-γ (1:300; cat^#^DF6045, Affinity Biosciences) and GOLM1 (1:300; cat^#^DF8100, Affinity Biosciences)] at 37°C for 1 h. Subsequently, after washing three times with the washing solution, the sections were incubated with biotinylated secondary antibody (1:200) for 30 min, followed by adding horseradish peroxidase-labeled streptavidin to the sections for 30 min. Finally, diaminobenzidine staining was used to detect the immunoreactivity of those tissues. The detailed procedures of H-score counting were demonstrated in our previous publication (Yin et al., 2019).

### Statistical analyses

We performed all the statistical analyses *via* R version 4.0.2. For the continuous data, a *t* test was used for comparison if the data is normally distributed; otherwise, the Wilcoxon rank-sum test was conducted. Categorical data were compared by Fisher’s exact test and the Chi-square test. Cox proportional hazard regression for hazard ratio (HR) and a log-rank test Kaplan–Meier curve, with the 95% confidential interval (95% CI), was performed to illustrate the difference in OS. The prediction efficiency of GOLM1 expression for OS was examined based on the time-dependent incident dynamic receiver operating characteristic (ROC) area that was below the curve (AUC) values (having a 60-month survival endpoint) (Blanche et al., 2013). Multivariate Cox regression analyses explored the prognostic value of GOLM1 after clinical feature adjustment. A two-sided *p*-value <0.05 demonstrated statistical significance.

## Results

### GOLM1 acts as an oncogene across cancers

**FIGURE 1:**
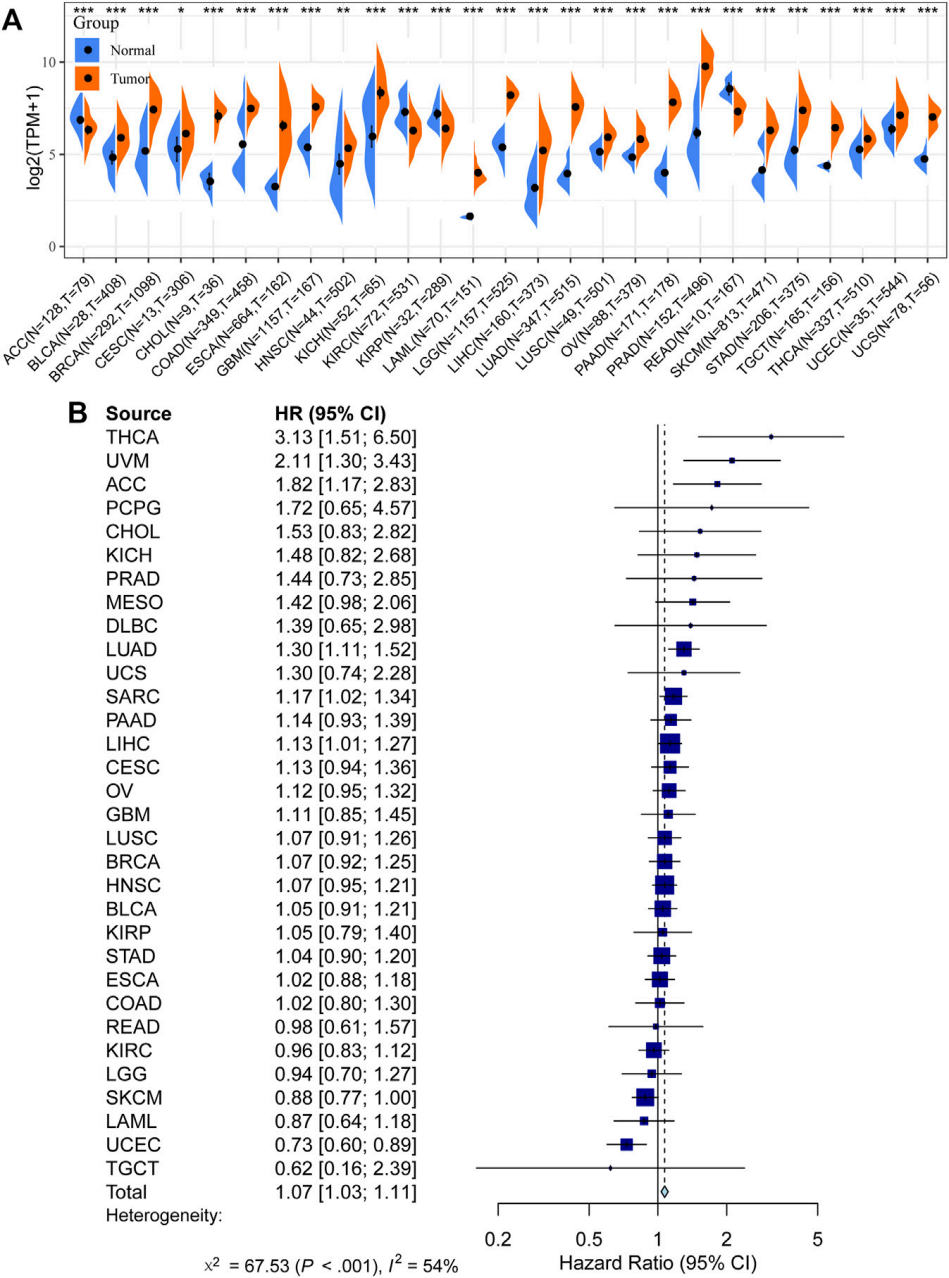
Prognostic value of GOLM1 across cancers. **(A)** Differential expression of GOLM1 in normal and tumor tissues; **(B)** Prognostic value of GOLM1 for overall survival across cancers.

We first compared the expression of GOLM1 in tumor and normal tissues. Tumor tissues were obtained from the TCGA project, while normal tissues combined tumor-adjacent tissues from the TCGA project and normal tissues from the GTEx project. Higher expression of GOLM1 in tumor tissues than in normal tissues was observed in most tumor types (*p* < 0.05, 85.19%, 23/27, Figure 1A). Furthermore, GOLM1 acted as an oncogene linked with poor prognosis in the combined meta-analysis of 32 types of cancer (*p* < 0.001, HR: 2.11, 95% CI: 1.30–3.43, Figure 1B).

### GOLM1 predicts the prognosis of uveal melanoma patients

**FIGURE 2:**
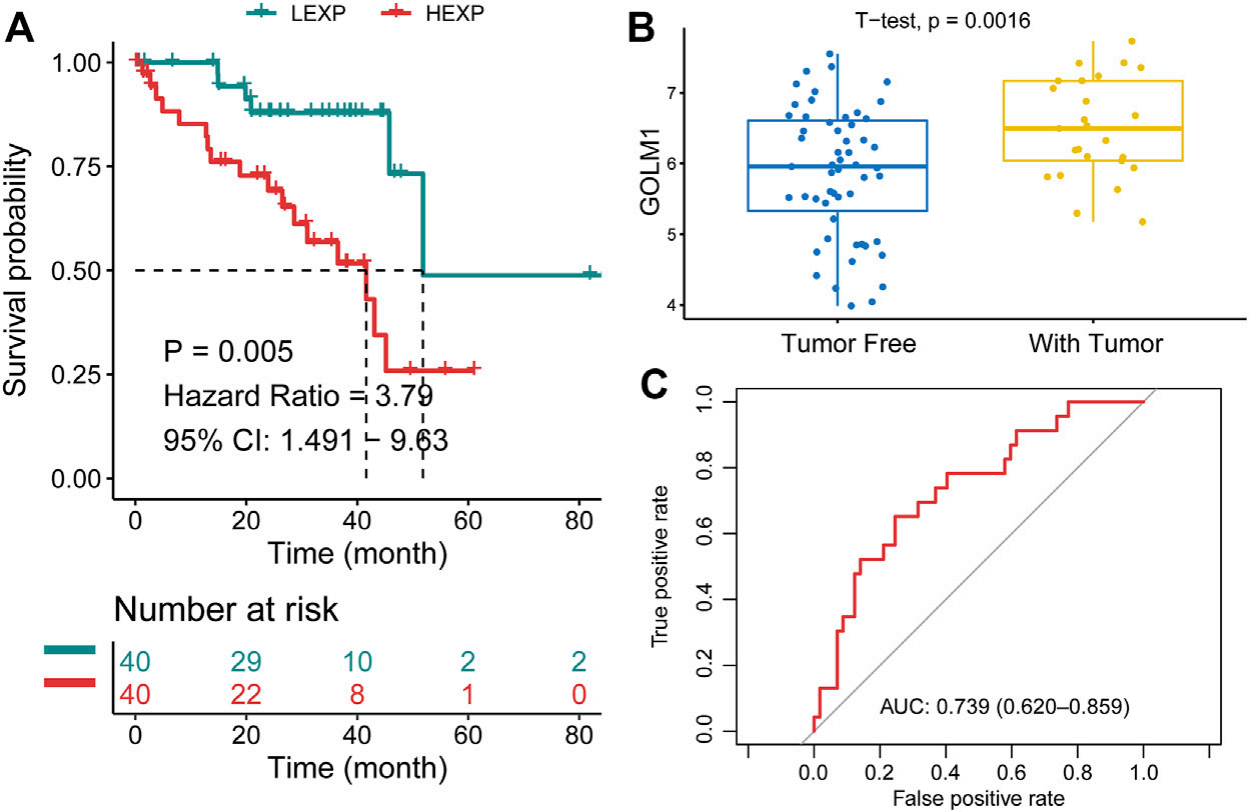
Prognostic value of GOLM1 in uveal melanoma. **(A)** Kaplan–Meier curve showing the polarized overall survival in GOLM1-low (LEXP) and GOLM1-high (HEXP) subgroups; **(B)** Differential expression of GOLM1 in patients lived with or without tumors; **(C)** Receiver operating characteristic curve showing the prognostic value of GOLM1.

In the TCGA-UVM cohort, we assigned patients to GOLM1-low (LEXP) and GOLM1-high (HEXP) subgroups, of which the latter subgroup presented poor prognosis (*p* = 0.005, HR: 3.790, 95% CI: 1.491–9.630, Figure 2A). The average OS time for HEXP patients was only 22.92 ± 17.33 months, while that of the other subgroup was 30.43 ± 18.26 months (*p* = 0.063, Table 2). Specifically, patients who lived with the tumor displayed a high level of GOLM1 compared to those without tumors (*p* = 0.0016, Figure 2B). Afterwards, we evaluated the prognostic value of GOLM1 over time, obtaining the AUC value of 0.739, which 95% CI ranged from 0.620 to 0.859 (Figure 2C), which represented a principal predictive value. After adjusting for age, sex, tumor stage, and tumor status, we revealed GOLM1 as an independent prognostic factor for UVM patients (*p* = 0.026, HR: 3.222, 95% CI: 1.152–9.010, Table 3).

**TABLE 2:** Summarization of the clinical features in TCGA-uveal melanoma cohort.

Clinical features	Level	GOLM1-low	GOLM1-high	*p*-value
Number		40	40	
Overall survival time (months)		30.43 ± 18.26	22.92 ± 17.33	0.063
Age (years)		59.00 ± 14.89	64.30 ± 12.56	0.089
Gender (%)	Female	18 (45.0)	17 (42.5)	1.000
	Male	22 (55.0)	23 (57.5)	
Stage (%)	Stage II	25 (62.5)	14 (35.0)	0.042*
	Stage III	14 (35.0)	22 (55.0)	
	Stage IV	1 (2.5)	3 (7.5)	
	Unknown	0 (0.0)	1 (2.5)	
Tumor status (%)	Tumor-free	31 (77.5)	24 (60.0)	0.148
	With tumor	9 (22.5)	16 (40.0)	
Basal diameter, cm		16.29 ± 3.23	17.58 ± 3.63	0.098
Thickness, cm		10.13 ± 2.50	10.71 ± 3.09	0.357
Tumor volume, cm^3^		756.77 ± 380.99	949.51 ± 576.01	0.085
Extrascleral extension (%)	No	37 (92.5)	31 (77.5)	0.124
	Yes	1 (2.5)	6 (15.0)	
	Unknown	2 (5.0)	3 (7.5)	

TCGA, the cancer genome atlas; *, *p* < 0.05.

**TABLE 3:** Multiple cox regression analysis results in two uveal melanoma cohorts.

Clinical features	HR	95% CI	*p*-value
TCGA-uveal melanoma			
Age, years	1.050	0.988–1.104	0.059
Gender, Male vs. Female	1.404	0.518–3.804	0.505
Stage, III vs. II	0.741	0.286–1.915	0.535
Stage, IV vs. II	24.025	1.965–293.782	0.013*
Tumor status, With tumor vs. Tumor-free	13.098	3.705–46.298	<0.001*
GOLM1, HEXP vs. LEXP	3.222	1.152–9.010	0.026*
GSE21138			
Age, years	1.040	1.003–1.08	0.036*
Gender, Male vs. Female	1.321	0.602–2.902	0.488
Extrascleral extension, Yes vs. No	2.438	0.883–6.727	0.085
GOLM1, HEXP vs. LEXP	2.681	1.220–5.888	0.014*
GSE84976			
Age, years	1.030	0.988–1.073	0.167
GOLM1, HEXP vs. LEXP	3.819	1.262–11.562	0.018*

TCGA, the cancer genome atlas; HR, hazard ratio; CI, confidential interval; *, *p* < 0.05.

### GOLM1 correlates with the tumor inflammatory/immune environment

**FIGURE 3:**
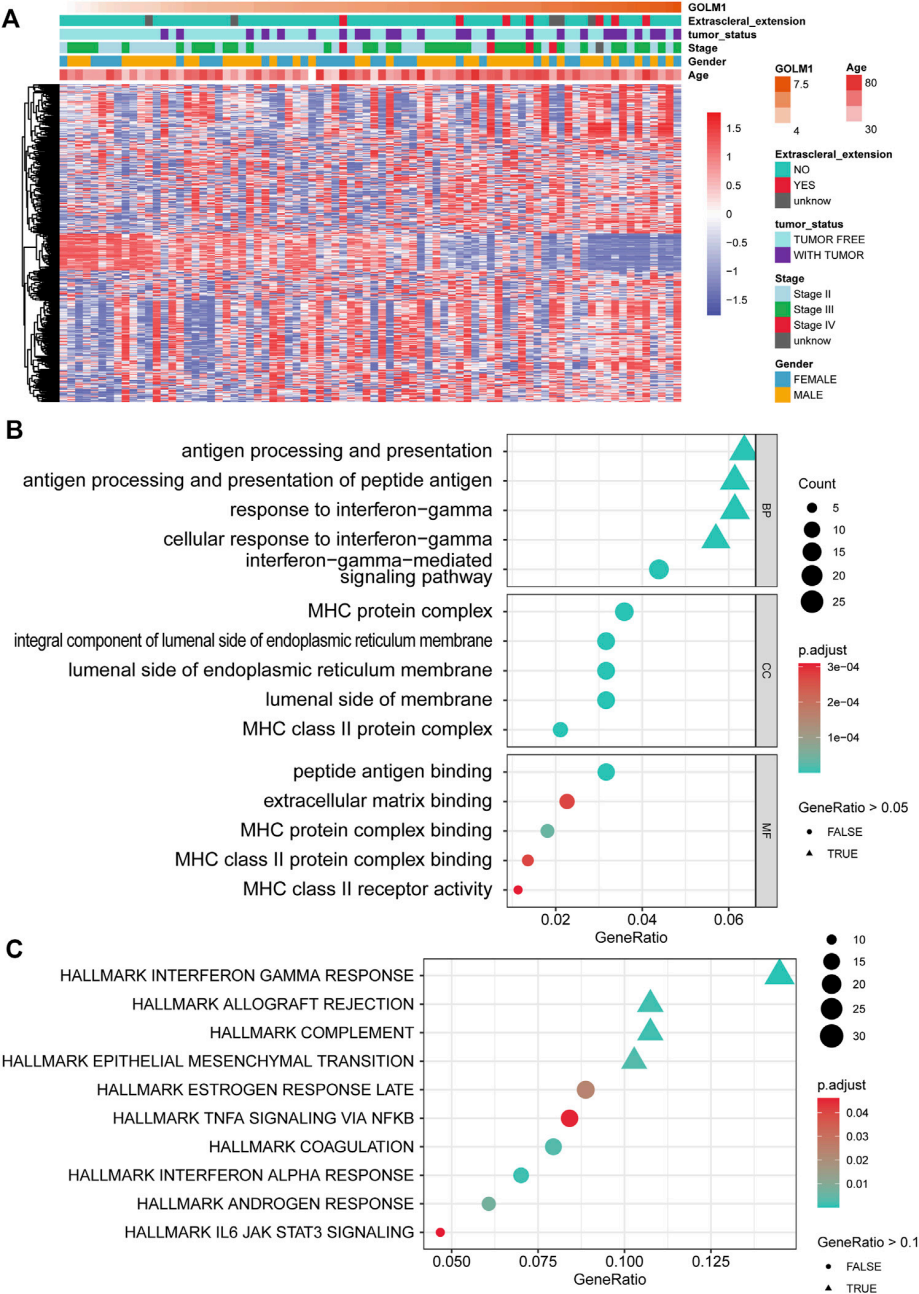
Pathway enrichment of GOLM1-associated genes. **(A)** Heatmap showing the expression of 531 differentially expressed genes (DEGs) between the GOLM1-low (LEXP) and GOLM1-high (HEXP) subgroups; **(B)** Pathway enrichment of the gene ontology (GO) terms for these DEGs; **(C)** Pathway enrichment of the HALLMARK terms for these DEGs.

To better understand how GOLM1 impacts tumor progression in UVM, efforts were made to search for potential interacting signaling pathways. First, we identified 531 DEGs between the LEXP and HEXP subgroups (Figure 3A), and the signaling pathways related to these DEGs were enriched. In the biological process analysis of GO terms, GOLM1 was mostly associated with the antigen processing and presentation pathway and the response to IFN-γ pathway; in the molecular function analysis, it exhibited associations with the MHC protein complex and the endoplasmic structure; in the cellular component analysis, GOLM1 influenced the binding of peptide antigen, extracellular matrix, and MHC protein complex (Figure 3B). And the pathway enrichment was also conducted based on the 50 classical HALLMARK tumor pathways, we observed the activation of interferon gamma response, allograft rejection, complement and epithelial mesenchymal transition (Figure 3C).

**FIGURE 4:**
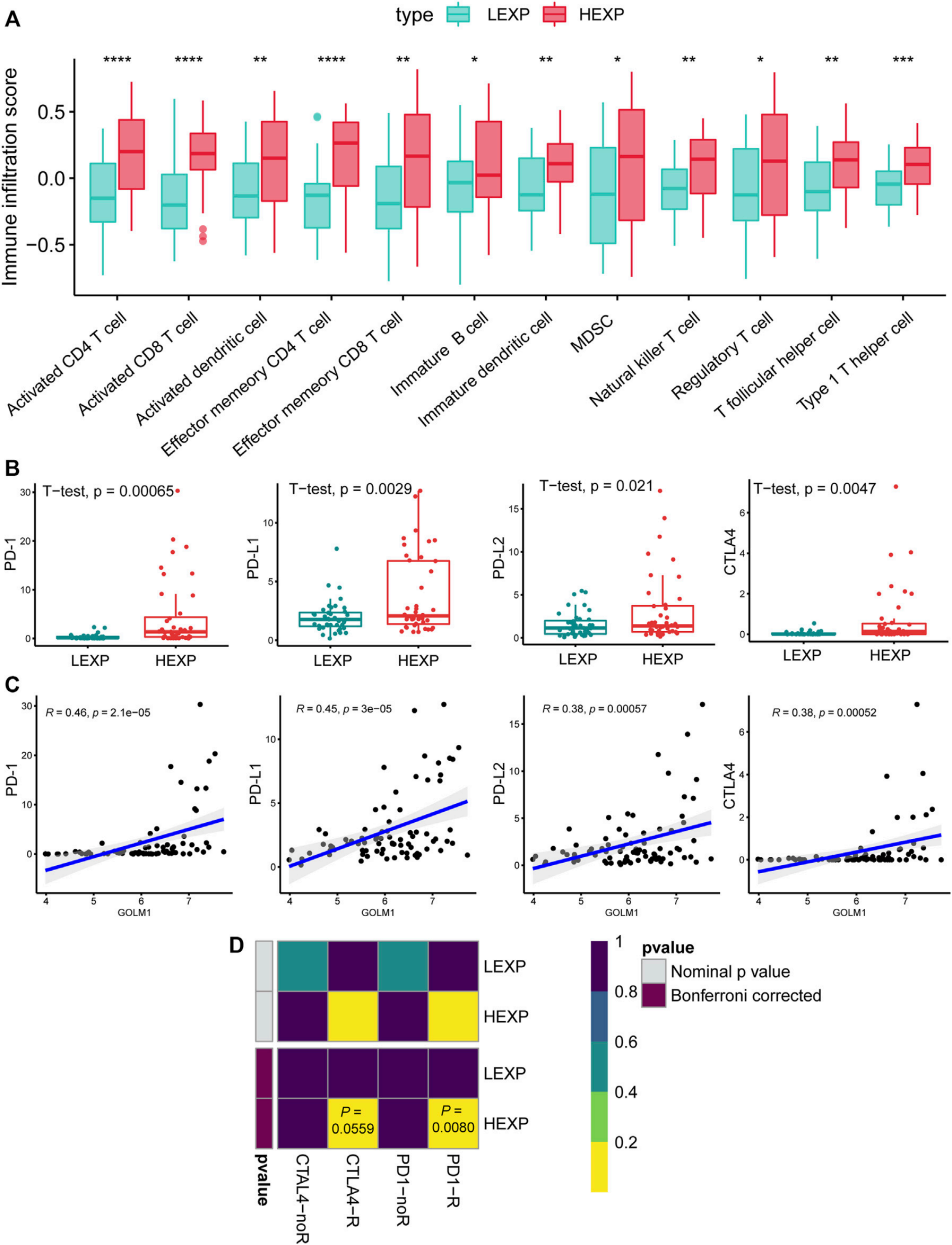
GOLM1 is linked with tumor-infiltrated immunocytes and immunotherapy. **(A)** Differential infiltration of immunocytes in the GOLM1-low (LEXP) group compared to the GOLM1-high (HEXP) group of The Cancer Genome Atlas (TCGA)-uveal melanoma cohort; **(B)** Differential expression of PD-1, PD-L1, PD-L2, and CTLA4 in the LEXP and HEXP subgroups of the TCGA-uveal melanoma cohort; **(C)** Correlation between GOLM1 and four immune checkpoints; **(D)** Prediction of the response to anti-CTLA4 and anti-PD-1/PD-L1 therapy.

These findings demonstrated the possible impact of GOLM1 on the immune infiltration of UVM. We further compared the infiltrated immunocytes in the LEXP and HEXP groups of GOLM1, of which the latter displayed a higher infiltration of type 1 T helper cells, CD8 T cells, natural killer T cells, T follicular helper cells, and CD4 T cells (all *p* < 0.05, Figure 4A).

### GOLM1 is positively associated with a favorable response to immunotherapy

Immune checkpoints are targets for immunotherapy in numerous cancers. Regarding the expression of the four main immune checkpoint genes, including PD-1, PD-L1, PD-L2, and CTLA4, the HEXP subgroup presented higher expression levels of these markers than LEXP (all *p* < 0.05, Figure 4B). In addition, the GOLM1 expression was positively correlated with the expression of PD-1 (*p* < 0.05, R = 0.46), PD-L1(*p* < 0.05, R = 0.45), PD-L2(*p* < 0.05, R = 0.38), and CTLA4(*p* < 0.05, R = 0.38) (Figure 4C). We also predicted the potential responders with the SubMap analysis of a melanoma cohort receiving anti-PD-1 and anti-CTLA4 therapies, demonstrating that patients in the HEXP group are more likely to be sensitive to both anti-CTLA4 and anti-PD-1/PD-L1 therapies (Figure 4D).

### External validation of GOLM1 prognostic value

**FIGURE 5:**
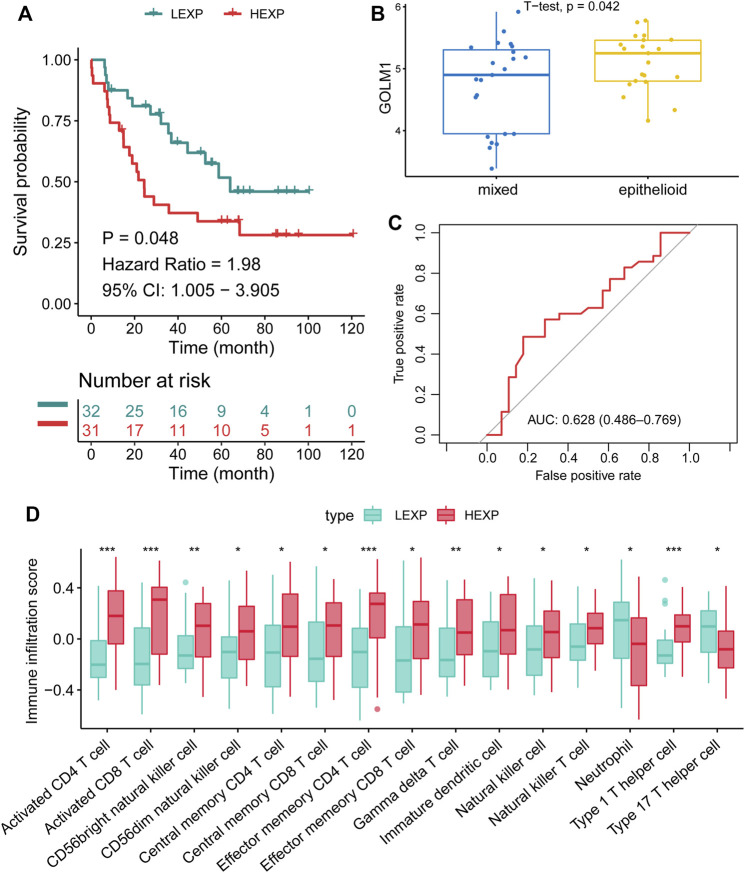
Confirmation of the prognostic value of GOLM1 in the GSE21138 cohort. **(A)** Kaplan–Meier curve showing the polarized overall survival in GOLM1-low (LEXP) and GOLM1-high (HEXP) subgroups; **(B)** Differential expression of GOLM1 in epithelioid tumors and mixed tumors; **(C)** Receiver operating characteristic curve showing the prognostic value of GOLM1; **(D)** Differential infiltration of immunocytes in the LEXP and HEXP groups.

To further confirm the prognostic value of GOLM1, we validated these findings in the GSE21138 cohort. Sixty-three patients were separated into the LEXP (*n* = 32) and HEXP (*n* = 31) subgroups based on the median value of GOLM1 expression. Patients in the HEXP subgroup displayed a 1.98-fold HR compared with the LEXP subgroup, with a 95% CI of 1.005–3.905 (*p* = 0.048, Figure 5A). The higher expression of GOLM1 in the epithelioid tumor than in the mixed tumor (*p* = 0.042, Figure 5B) was consistent with the worst prognosis of epithelioid UVM. Based on the adjustment of age, sex, and extrascleral extension, we revealed GOLM1 to be an independent prognostic factor (*p* = 0.014, HR: 2.681, 95% CI: 1.220–5.888, Table 3). The AUC of GOLM1 in this cohort is 0.628 (95% CI: 0.486–0.769, Figure 5C). In the comparison of tumor-infiltrated immunocytes, the HEXP group of the GSE21138 cohort presented higher infiltrations, especially in the T cells (Figure 5D).

**FIGURE 6:**
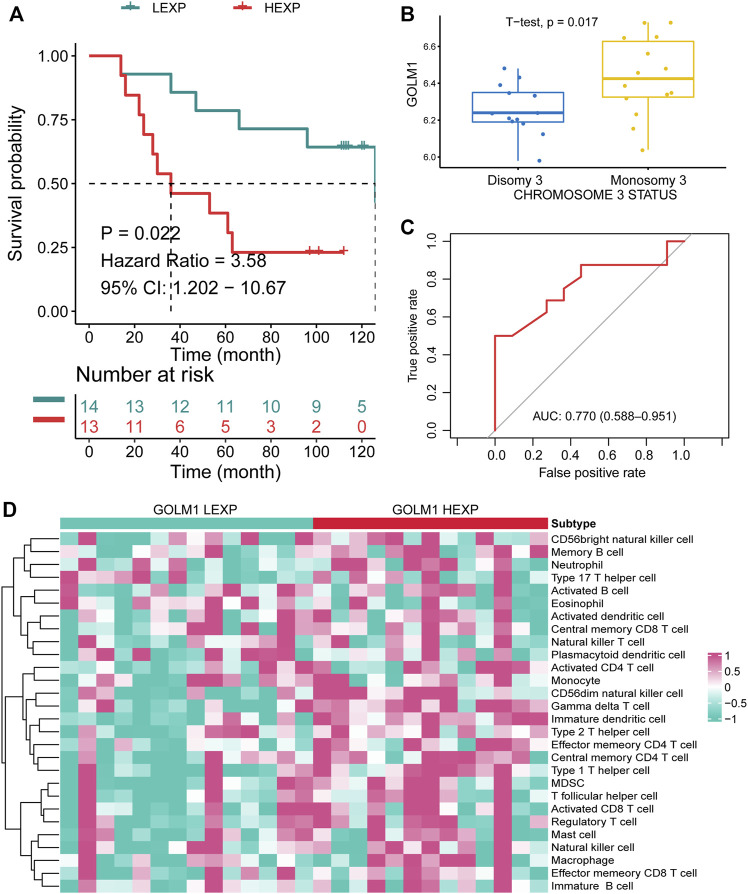
Confirmation of the prognostic value of GOLM1 in the GSE84976 cohort. **(A)** Kaplan–Meier curve showing the polarized overall survival in GOLM1-low (LEXP) and GOLM1-high (HEXP) subgroups; **(B)** Differential expression of GOLM1 in patients with disomy and monosomy chromosome 3; **(C)** Receiver operating characteristic curve showing the prognostic value of GOLM1; **(D)** Differential infiltration of immunocytes in the LEXP and HEXP groups.

As for GSE84976 cohort, 27 patients were separated into the LEXP (*n* = 14) and HEXP (*n* = 13) subgroups based on the median value of GOLM1 expression. Patients in the HEXP subgroup displayed a 3.58-fold HR compared with the LEXP subgroup, with a 95% CI of 1.202–10.67 (*p* = 0.022, Figure 6A). Monosomy chromosome 3 indicates the poor prognosis of UVM as widely reported, and we observed that patients with monosomy chromosome 3 contained the higher level of GOLM1 than patients with disomy chromosome 3 (*p* = 0.017, Figure 6B), which consistent with the above findings. The AUC of GOLM1 in this cohort was 0.770, with 95% CI ranged from 0.588 to 0.951, demonstrating a moderate prognostic value (Figure 6C). We also observed the enrichment of immunocytes infiltration in GOLM1 HEXP patients in GSE84976 cohort, especially gamma delta T cell, memory B cell and immature dendritic cell (Figure 6D).

### Validation of the links between GOLM1 and immune markers by IHC

**FIGURE 7:**
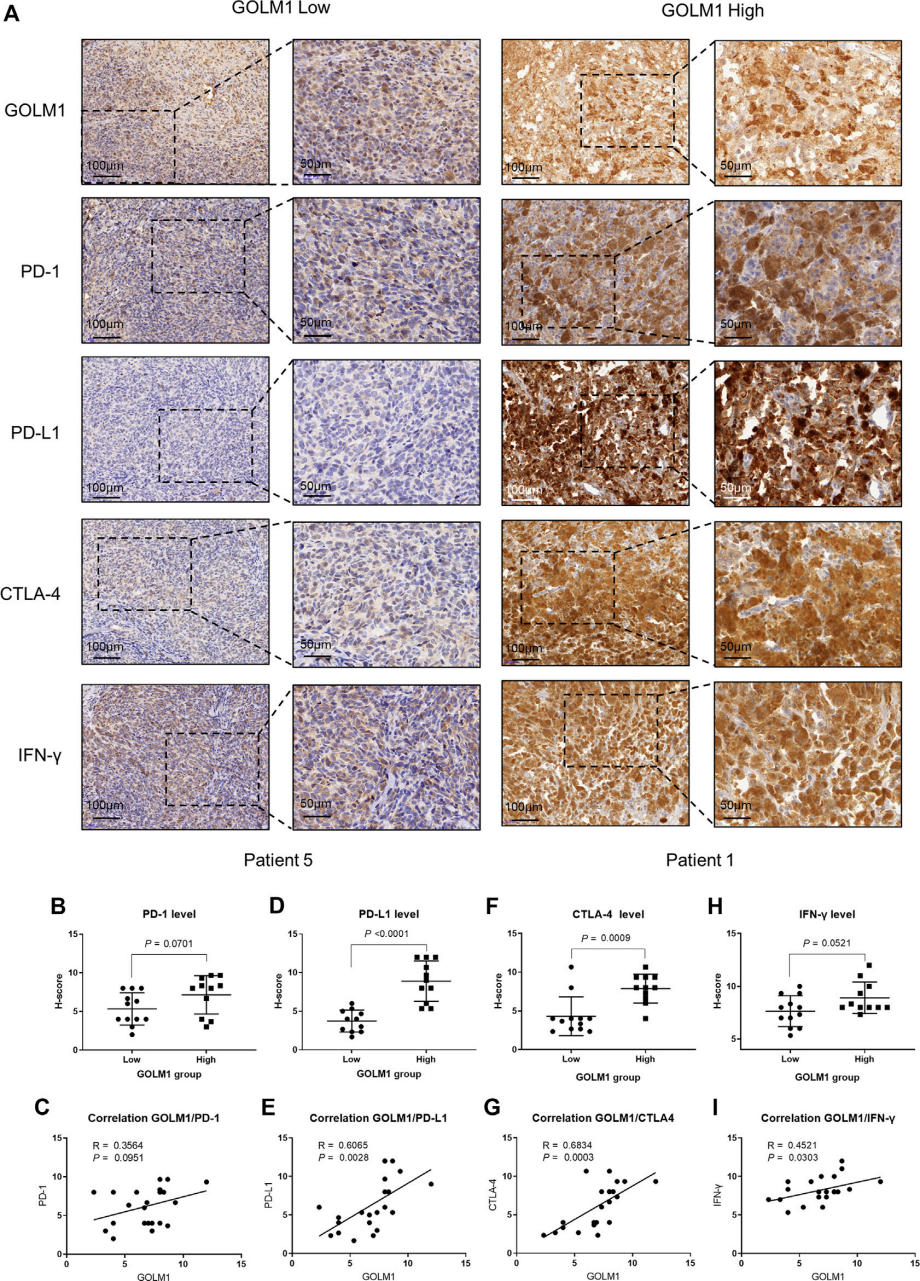
Correlation between GOLM1 expression and PD-1, PD-L1, CTLA4, and IFN-γ. **(A)** Immunohistochemistry assay revealed the protein expression of GOLM1, PD-1, PD-L1, CTLA4, and IFN-γ. **(B,C)** The expression difference of PD-1 among GOLM1-low (LEXP) and GOLM1-high (HEXP) subgroups **(B)** and the correlation between PD-1 and GOLM1 expression **(C)**. **(D,E)** The expression difference of PD-L1 among the LEXP and HEXP groups **(D)** and the correlation between PD-L1 and GOLM1 expression **(E)**. **(F,G)** The expression difference of CTLA4 among the LEXP and HEXP groups **(F)** and the correlation between CTLA4 and GOLM1 expression **(G)**. **(H,I)** The expression difference of IFN-γ among the LEXP and HEXP groups **(H)** and the correlation between IFN-γ and GOLM1 expression **(I)**.

As mentioned above, we revealed the links between GOLM1 and immune checkpoints, as well as immunocytes. We collected 23 tumor cases and evaluated the protein levels of GOLM1, PD-1, PD-L1, CTLA4, and IFN-γ by IHC staining. All the original pictures were put in Supplementary Figure S1, and two representative patients were chosen to display in Figure 7A, patient 1 with positive GOLM1 and patient 5 with negative GOLM1. We first separated the 23 patients into LEXP (*n* = 12) and HEXP (*n* = 11) subgroups according to the H-score. We found that patients belonging to the HEXP subgroup also displayed high levels of PD-1 (*p* = 0.0701, Figure 7B), PD-L1 (*p* < 0.001, Figure 7D), CTLA-4 (*p* = 0.009, Figure 7F), and IFN-γ (*p* = 0.0521, Figure 7H) compared to those with LEXP. Moreover, the increased H-score of GOLM1 was obviously positively correlated with the elevated values of PD-1 (R = 0.3564, *p* = 0.0951, Figure 7C), PD-L1 (R = 0.6065, *p* = 0.0028, Figure 7E), CTLA-4 (R = 0.6834, *p* = 0.0003, Figure 7G) and IFN-γ (R = 0.4521, *p* = 0.0303, Figure 7I). These results from the IHC staining not only supported the new findings revealed in the current study but also suggested that these patients with a high level of GOLM1 commonly had an elevated abundance of immune checkpoint markers, highlighting its potential role in serving as a therapeutic target or immunotherapy indicator.

## Discussion

To identify novel prognostic biomarkers that could be used to reflect the tumor-infiltrated inflammatory/immune microenvironment, we performed the current study. As a result, we found that GOLM1 was able to predict the overall survival outcomes of UVM patients and served as a risk factor independent of clinicopathological features. We analyzed the co-expressed genes of GOLM1, and pathway analysis indicated that these genes mainly belonged to immune-related pathways. We also analyzed the tumor-infiltrated immunocyte proportion differences between the LEXP and HEXP subgroups, with significant variations identified. Tumor microenvironment activity is correlated with the efficacy of immune checkpoint blockade therapy. We performed SubMap analysis and revealed that the UVM patients in the HEXP group were predicted to be more sensitive to PD-1/PD-L1 and CTLA4 therapies than those in the LEXP subgroup. Our findings suggested that GOLM1 was a novel prognostic marker for UVM patients and could be employed to predict immunotherapy treatment efficacy.

The prognostic value of GOLM1 was first tested at the pancancer level, the result of which presented the highest predictive value in thyroid carcinoma, followed by UVM. Upregulation of GOLM1 was observed in multiple cancers and is involved in the mediation of malignant behaviors. Such upregulation in prostate cancer promoted the proliferation and progression of cancer cells by activating PI3K/AKT/mTOR signaling (Yan et al., 2018), thereby being recognized as a biomarker in detecting prostate cancer and evaluating its aggressiveness (Varambally et al., 2008; Li et al., 2012). Similar findings were obtained in hepatocellular carcinoma. Chen et al. (2015) found that abnormal expression of GOLM1 increased the proliferation and progression of hepatocellular carcinoma both *in vitro* and *in vivo*. Furthermore, the prognostic value of GOLM1 in predicting overall survival and recurrence-free survival among hepatocellular carcinoma patients was also proven (Mao et al., 2010; Bao et al., 2013). Moreover, the oncogenic role and prognostic value of GOLM1 in non-small-cell lung cancer were identified (Zhang et al., 2010; Zhang et al., 2017b). Given the importance of GOLM1 in cancers, its prognostic value, functional role, and potential mechanisms in UVM have aroused wide research interest. In addition to its prognostic value, we performed extensive analyses to reveal the underlying mechanisms regarding the worse survival outcomes of the HEXP subgroup than the LEXP subgroup. Notably, GOLM1 expression was found to be positively associated with the proportions of tumor-infiltrated immunocytes. Patients with higher GOLM1 expression were also predicted to be more sensitive to PD-1/PD-L1 and CTLA4 treatment than those with lower GOLM1 expression. For further validation, we performed an IHC assay and obtained consistent findings that the expression of GOLM1 was positively associated with the expression of PD-1, PD-L1, CTLA4, and IFN-γ. This result highlighted its potential role in guiding clinical immune checkpoint blockade therapy.

## Conclusion

In summary, we systematically analyzed the prognostic role of GOLM1 at the pan-cancer level and revealed its high predictive value for the overall survival of UVM. The results also identified it as a risk factor independent of clinicopathological features. The expression of this gene is positively associated with PD-1, PD-L1, CTLA4, and IFN-γ expression, and patients with higher expression of GOLM1 are predicted to be more sensitive to anti-CTLA4 as well as anti-PD-1/PD-L1 therapies. These findings offer individual clinical outcome predictions and immune checkpoint blockade therapy guidance for UVM patients.

## Data Availability

The original contributions presented in the study are included in the article/Supplementary Material, further inquiries can be directed to the corresponding author.
