# Supplementary material for: GOLM1 is related to the inflammatory/immune nature of uveal melanoma and acts as a promising indicator for prognosis and immunotherapy response
Source: Front Genet. 2022 Nov 18;13:1051168. doi: 10.3389/fgene.2022.1051168 (PMC9716024; doi:10.3389/fgene.2022.1051168)
Supplement: Supplementary file 1 [file Table1.DOCX]

**Supplementary Table 1** Baseline demographics and clinical characteristics of patients

| ID | Sex | Age at diagnosis | Eye side | Tumor event (metastasis or recurrence) | Survival status |
| --- | --- | --- | --- | --- | --- |
| 1 | Male | 49 | OD | Metastasis | DEAD |
| 2 | Female | 51 | OS | No | LIVE |
| 3 | Female | 76 | OS | Recurrence | N/A |
| 4 | Male | 60 | OS | N/A | DEAD |
| 5 | Female | 67 | OD | Recurrence | LIVE |
| 6 | Female | 85 | OS | N/A | N/A |
| 7 | Female | 51 | OS | N/A | N/A |
| 8 | Male | 77 | OS | No | LIVE |
| 9 | Female | 54 | OS | N/A | N/A |
| 10 | Male | 70 | OS | N/A | N/A |
| 11 | Male | 75 | OS | No | LIVE |
| 12 | Female | 50 | OS | N/A | N/A |
| 13 | Male | 67 | OD | No | LIVE |
| 14 | Female | 44 | OS | No | LIVE |
| 15 | Male | 60 | OS | N/A | DEAD |
| 16 | Male | 55 | OD | No | LIVE |
| 17 | Female | 44 | OS | N/A | N/A |
| 18 | Male | 45 | OD | N/A | DEAD |
| 19 | Female | 74 | OS | N/A | DEAD |
| 20 | Female | 39 | OD | Recurrence | N/A |
| 21 | Male | 53 | OS | Recurrence | LIVE |
| 22 | Female | 79 | OD | Recurrence | DEAD |
| 23 | Female | 79 | N/A | N/A | N/A |

Abbreviation: OD: oculus dexter; OS: oculus sinister; N/A: Not Applicable
