# Supplementary material for: GOLM1 is related to the inflammatory/immune nature of uveal melanoma and acts as a promising indicator for prognosis and immunotherapy response
Source: Front Genet. 2022 Nov 18;13:1051168. doi: 10.3389/fgene.2022.1051168 (PMC9716024; doi:10.3389/fgene.2022.1051168)

ID: 1

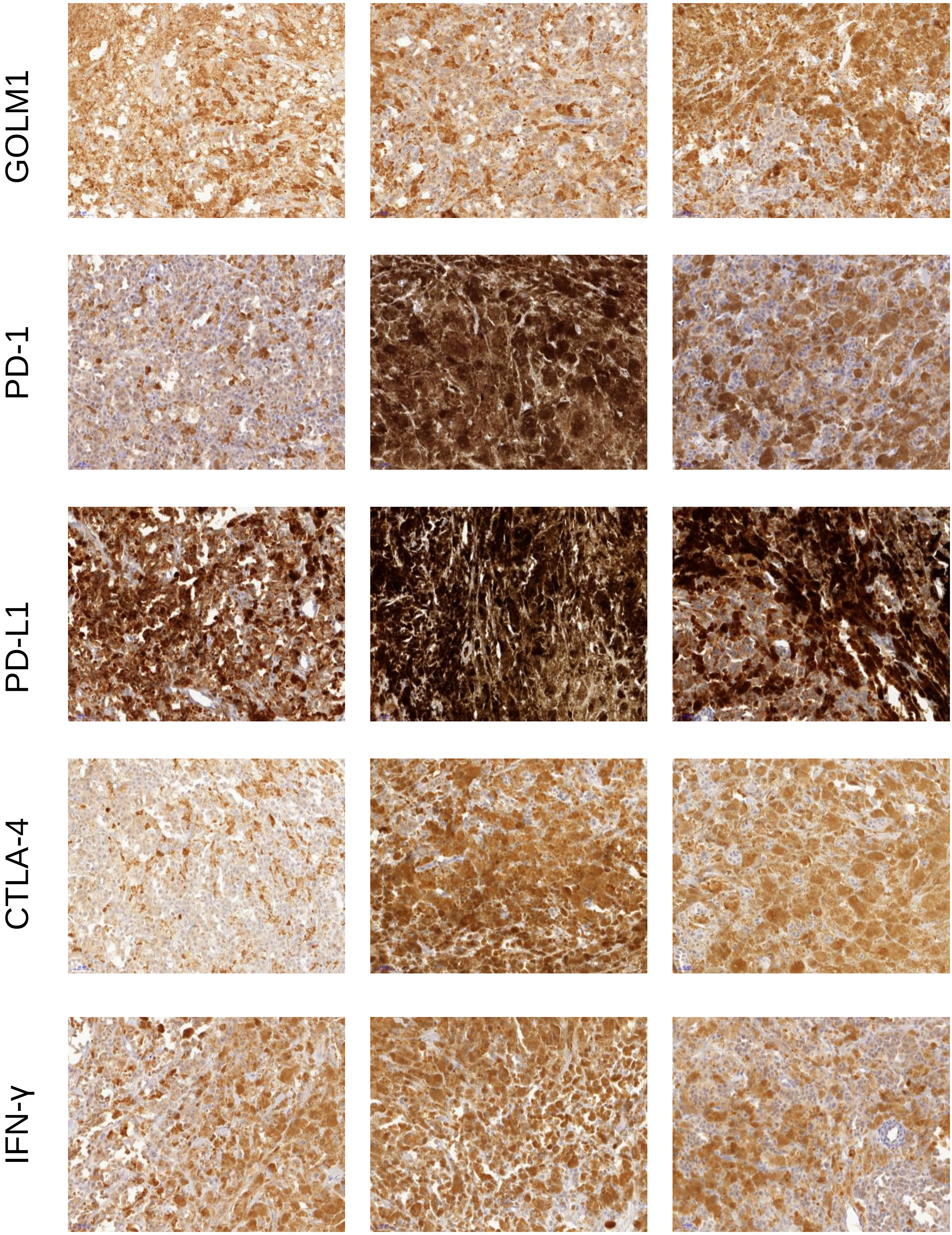

ID: 2

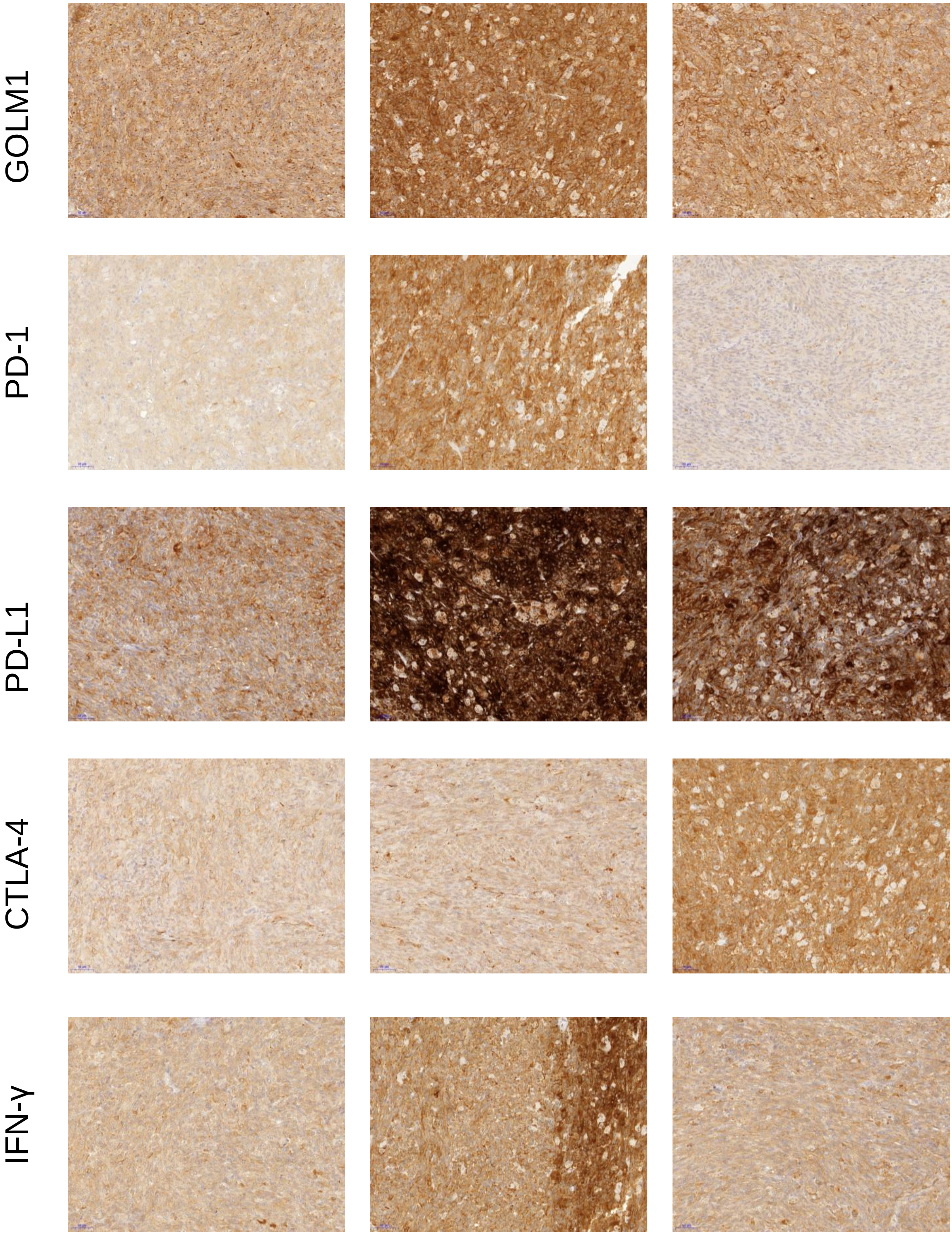

ID: 3

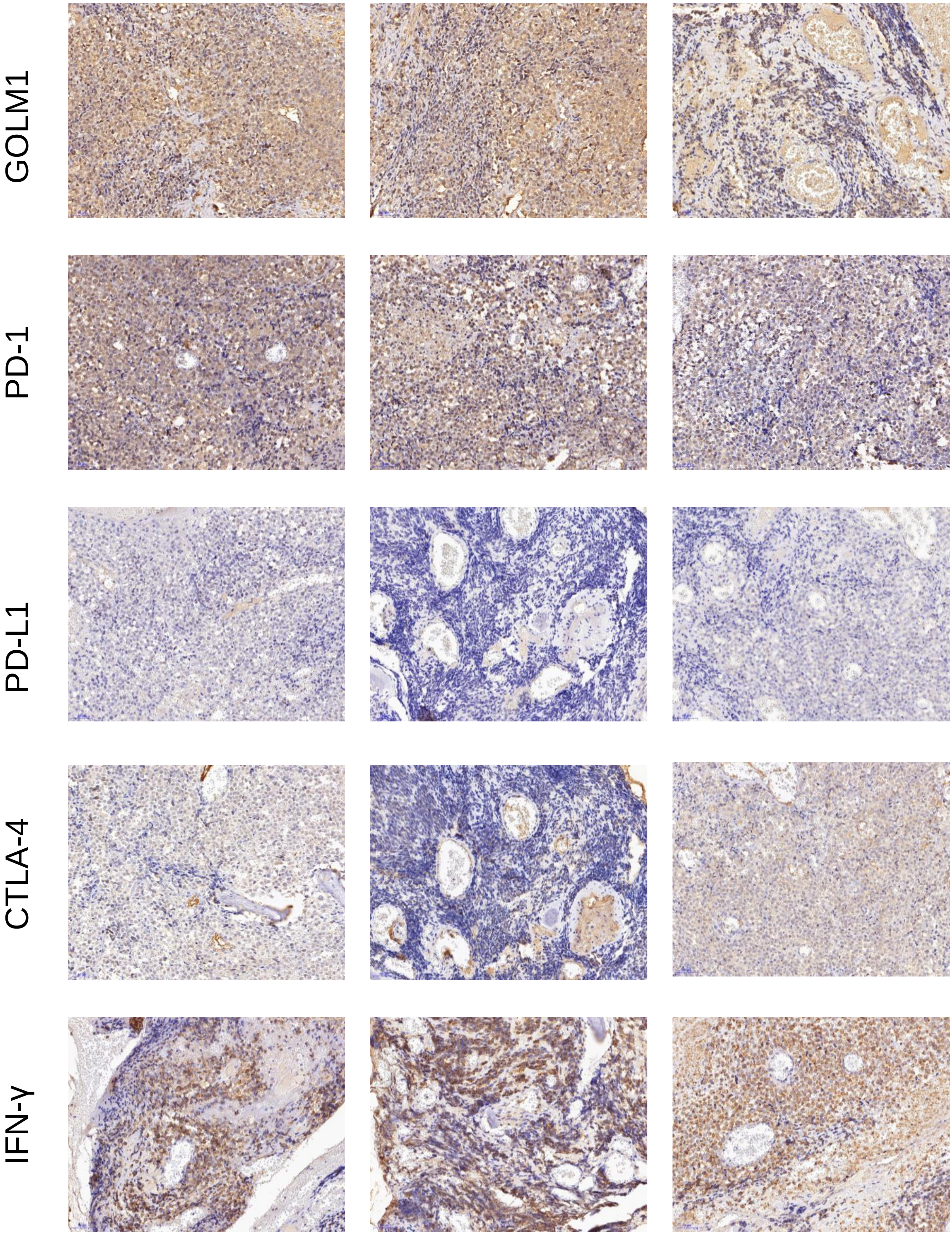

ID: 4

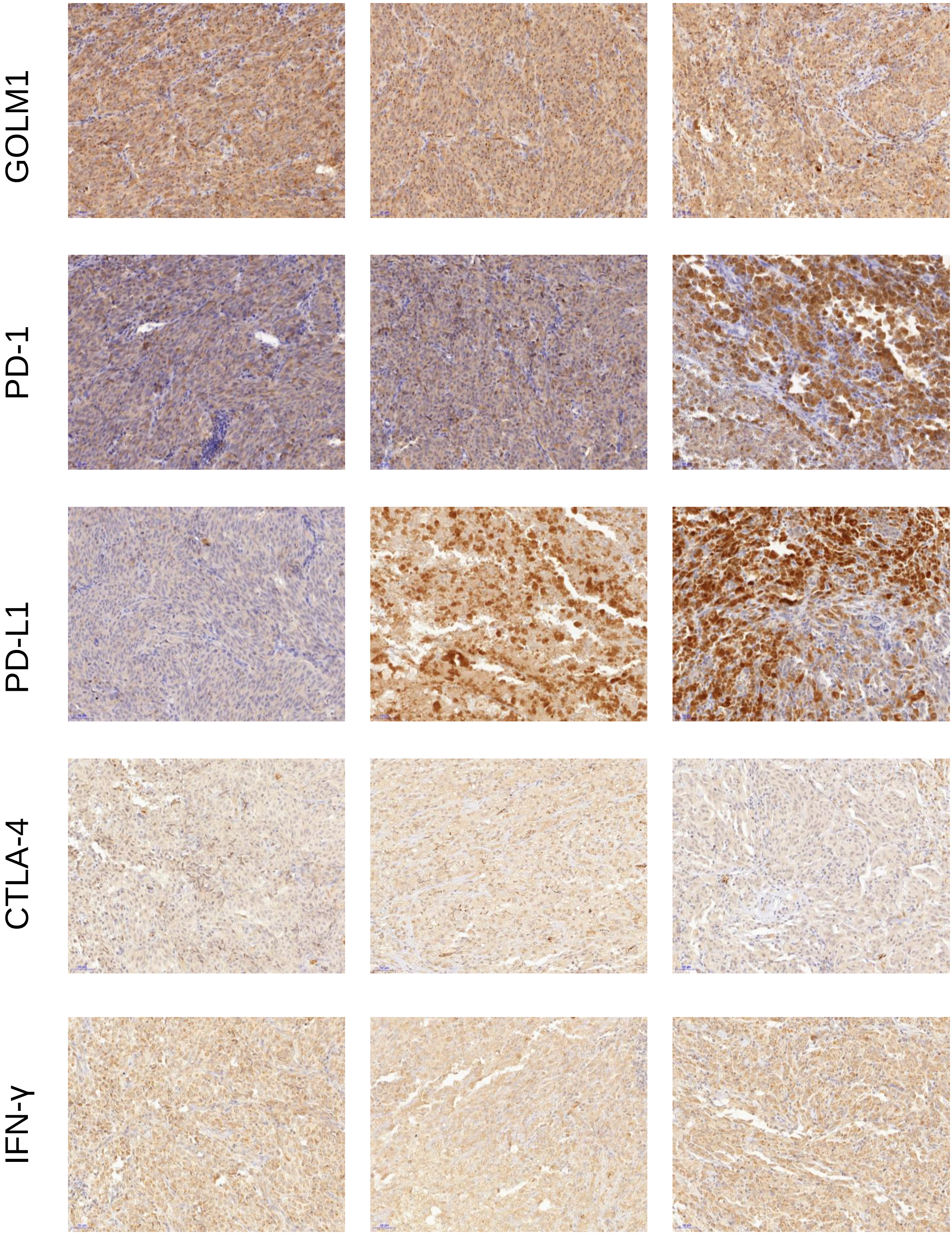

ID: 5

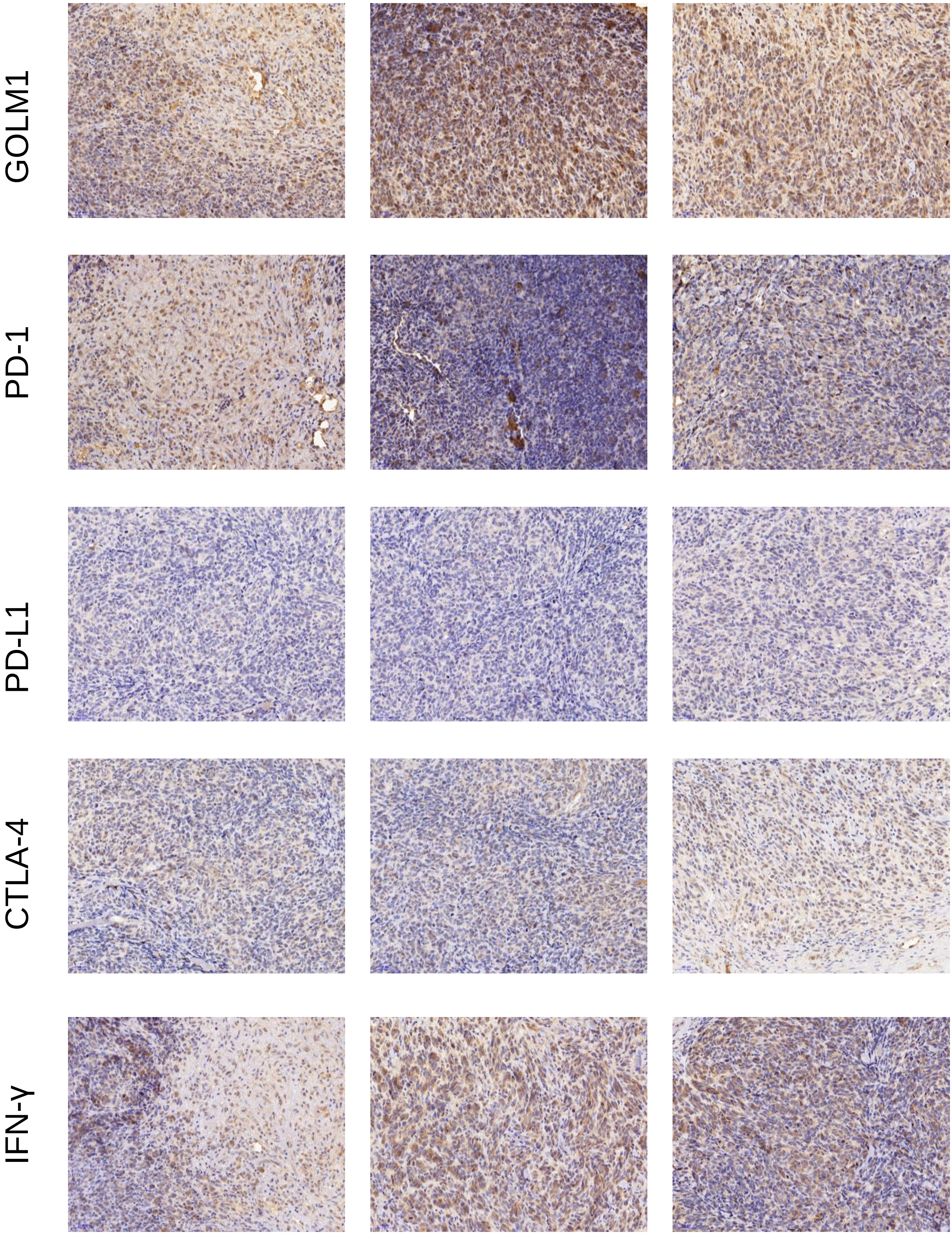

ID: 6

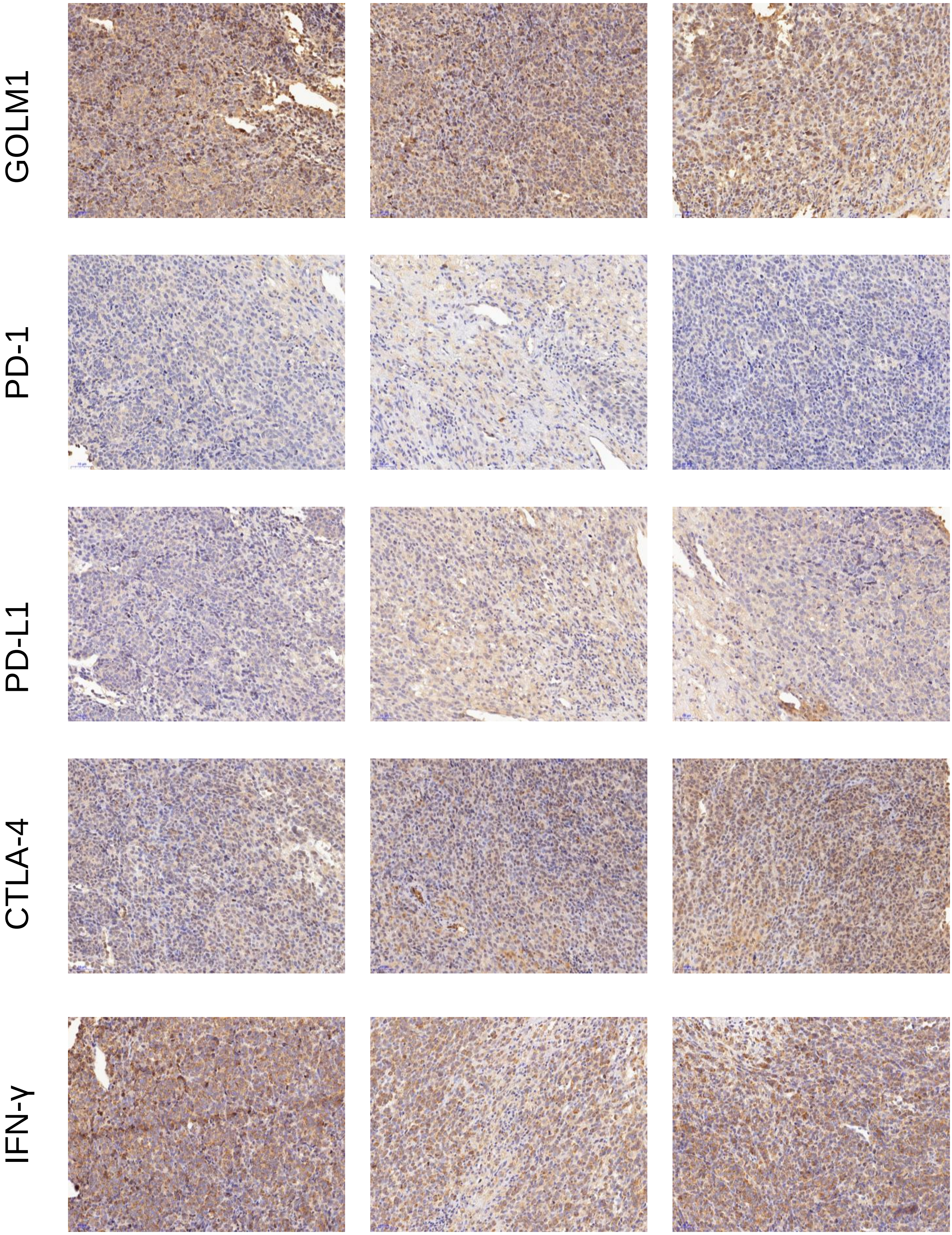

ID: 7

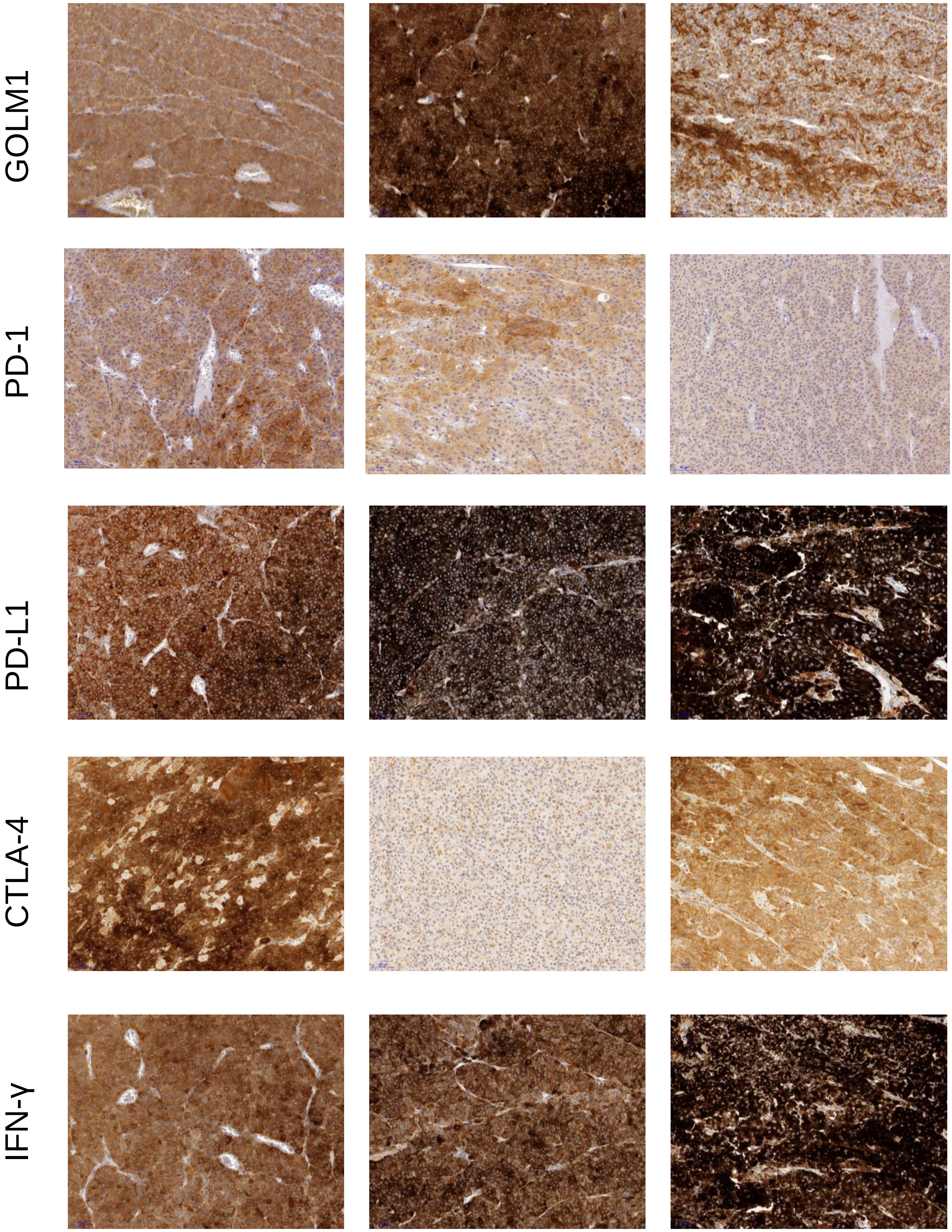

ID: 8

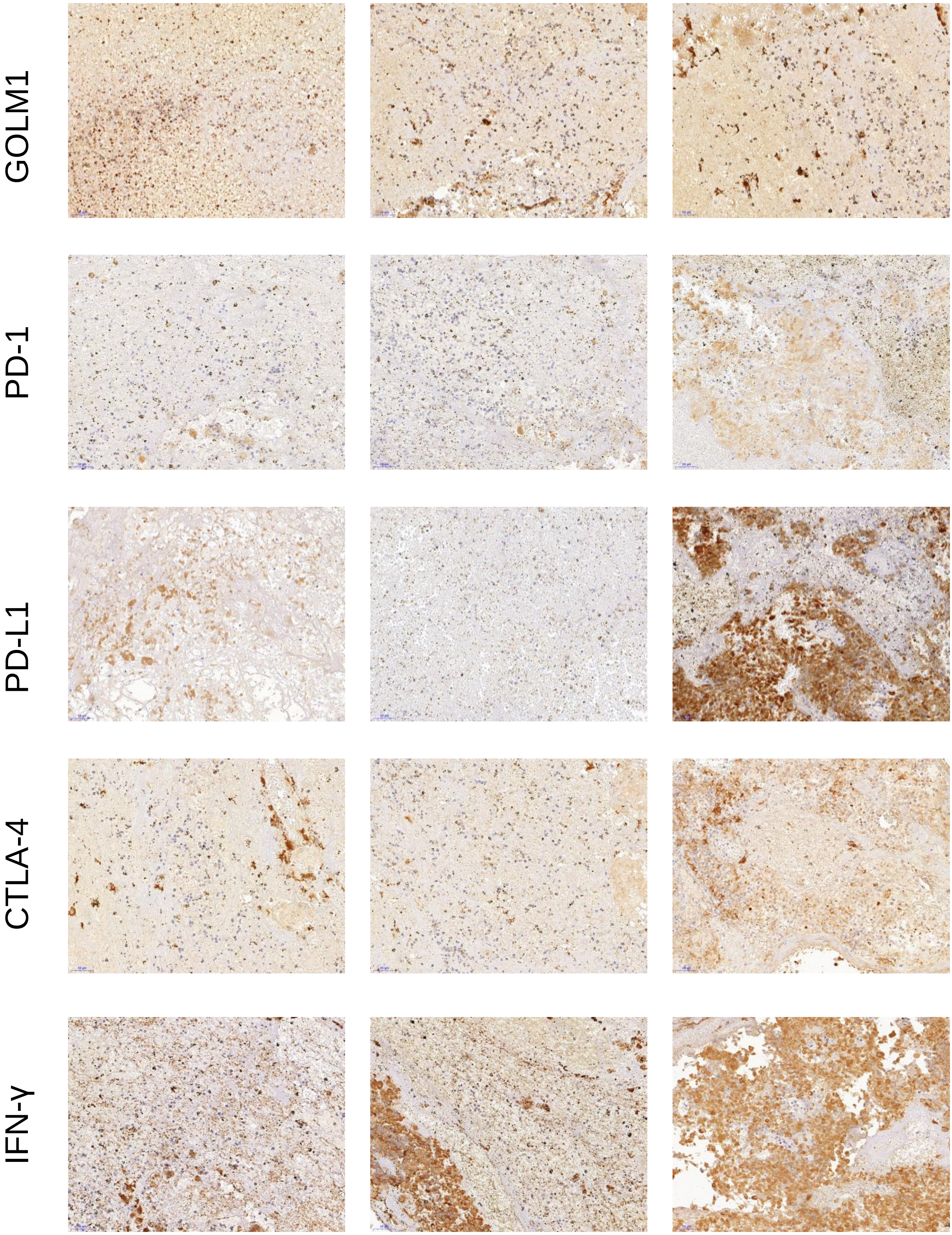

ID: 9

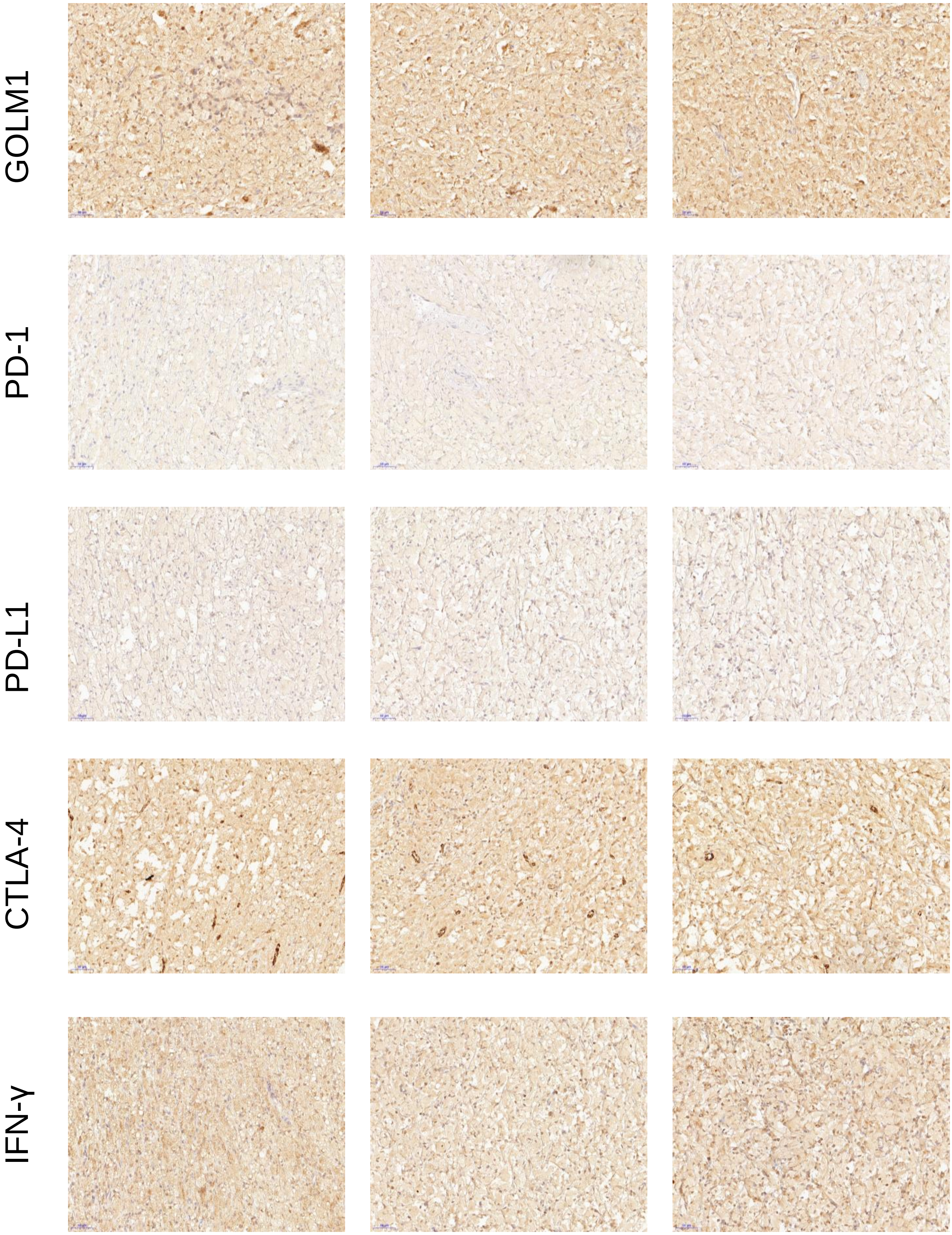

ID: 10

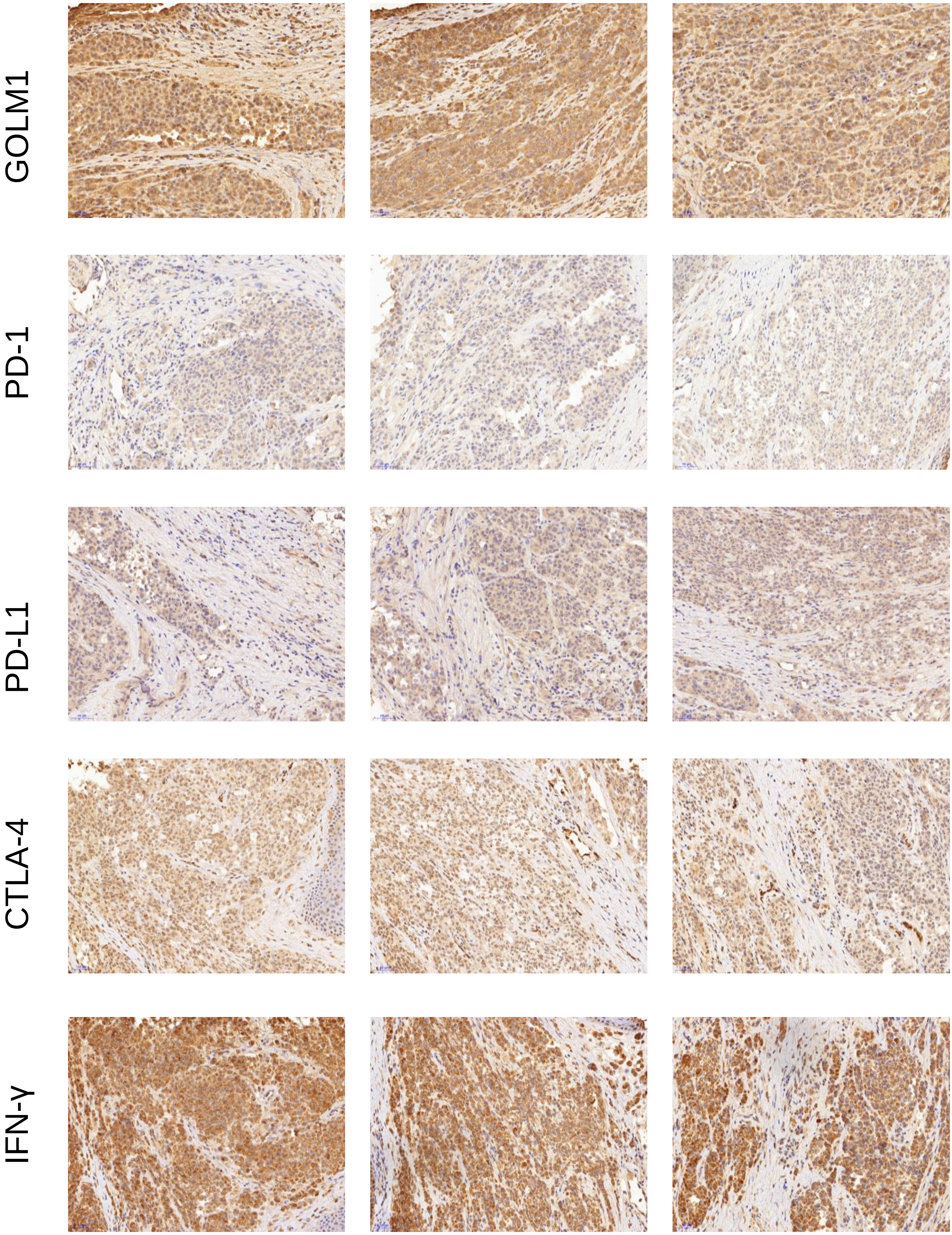

ID: 11

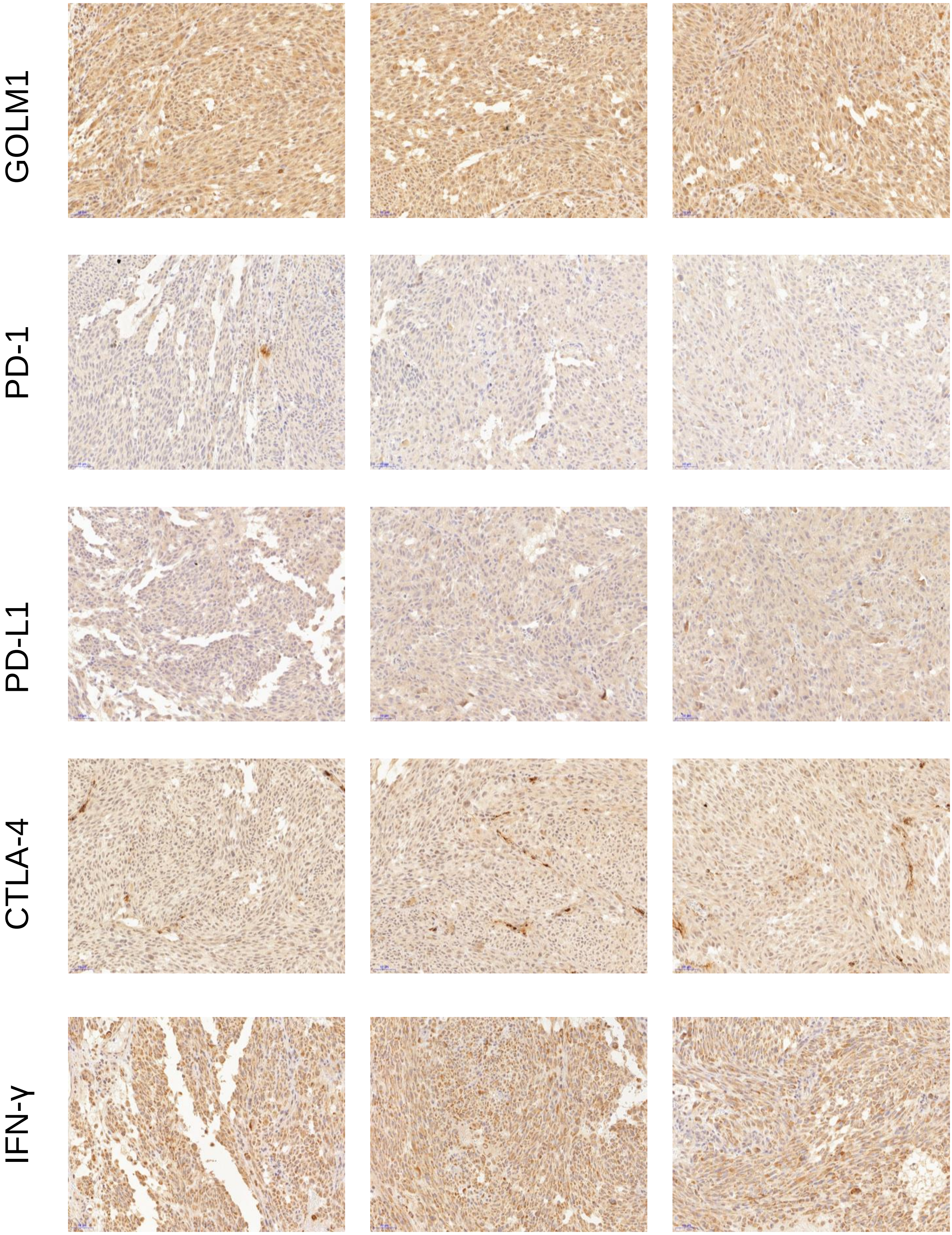

ID: 12

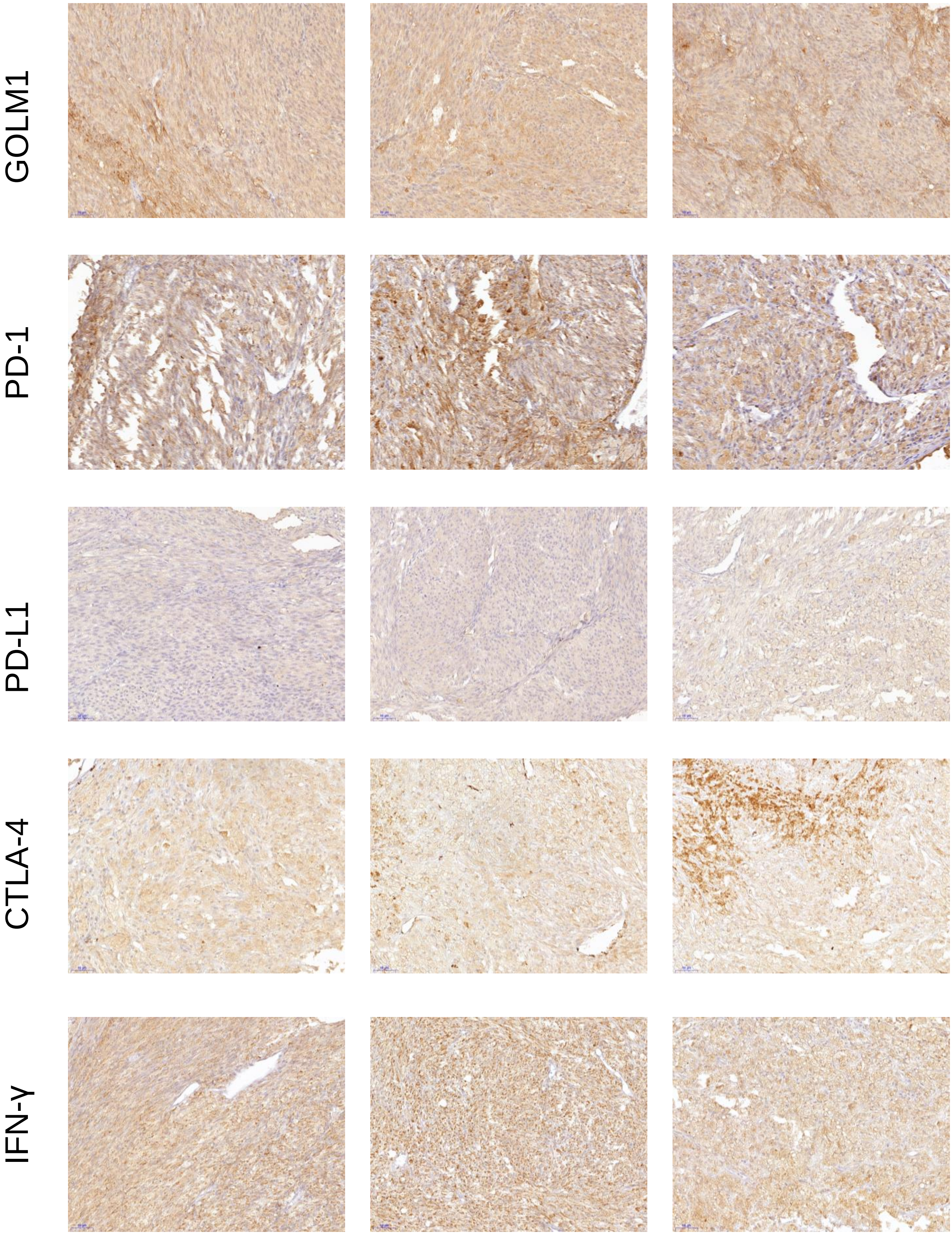

ID: 13

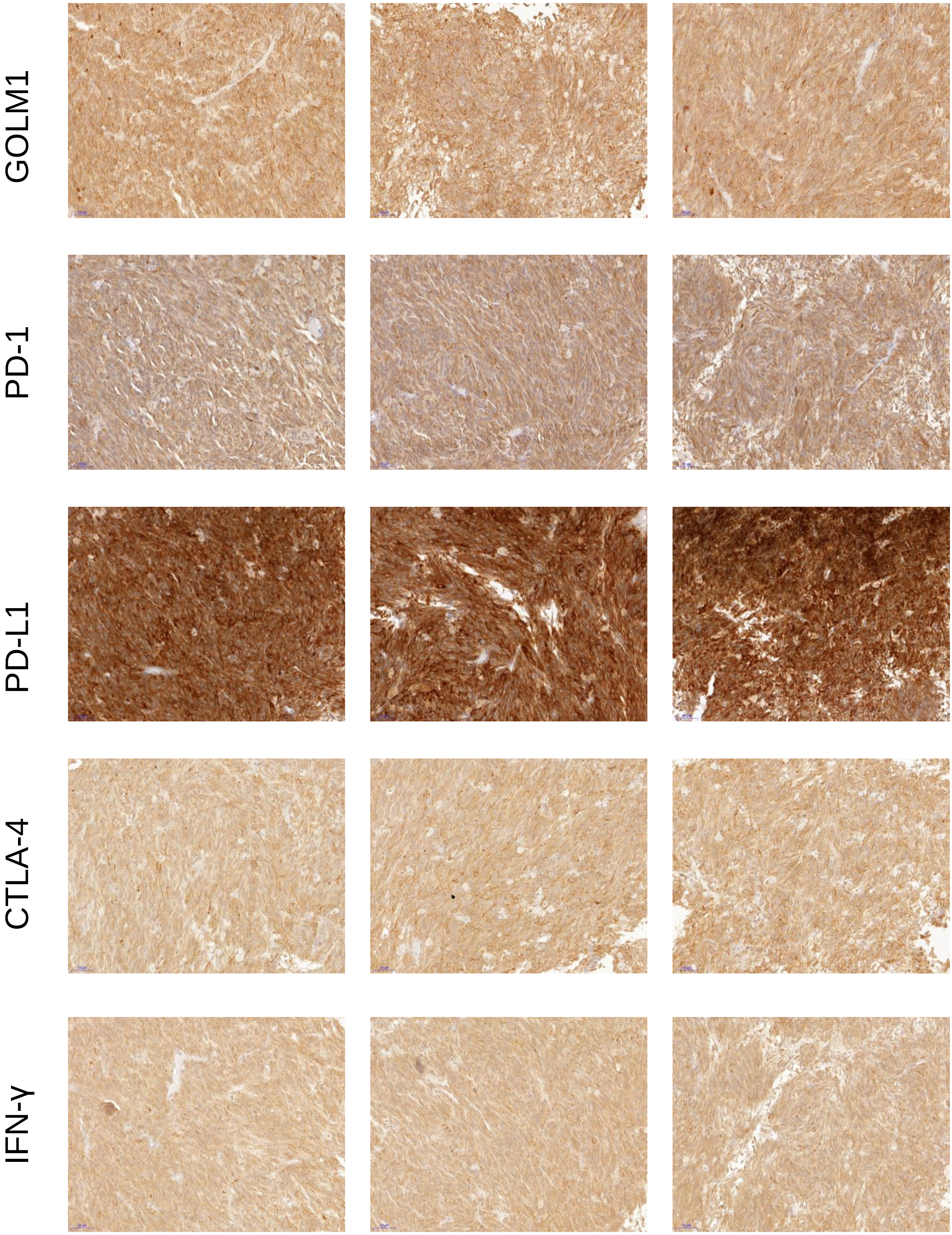

ID: 14

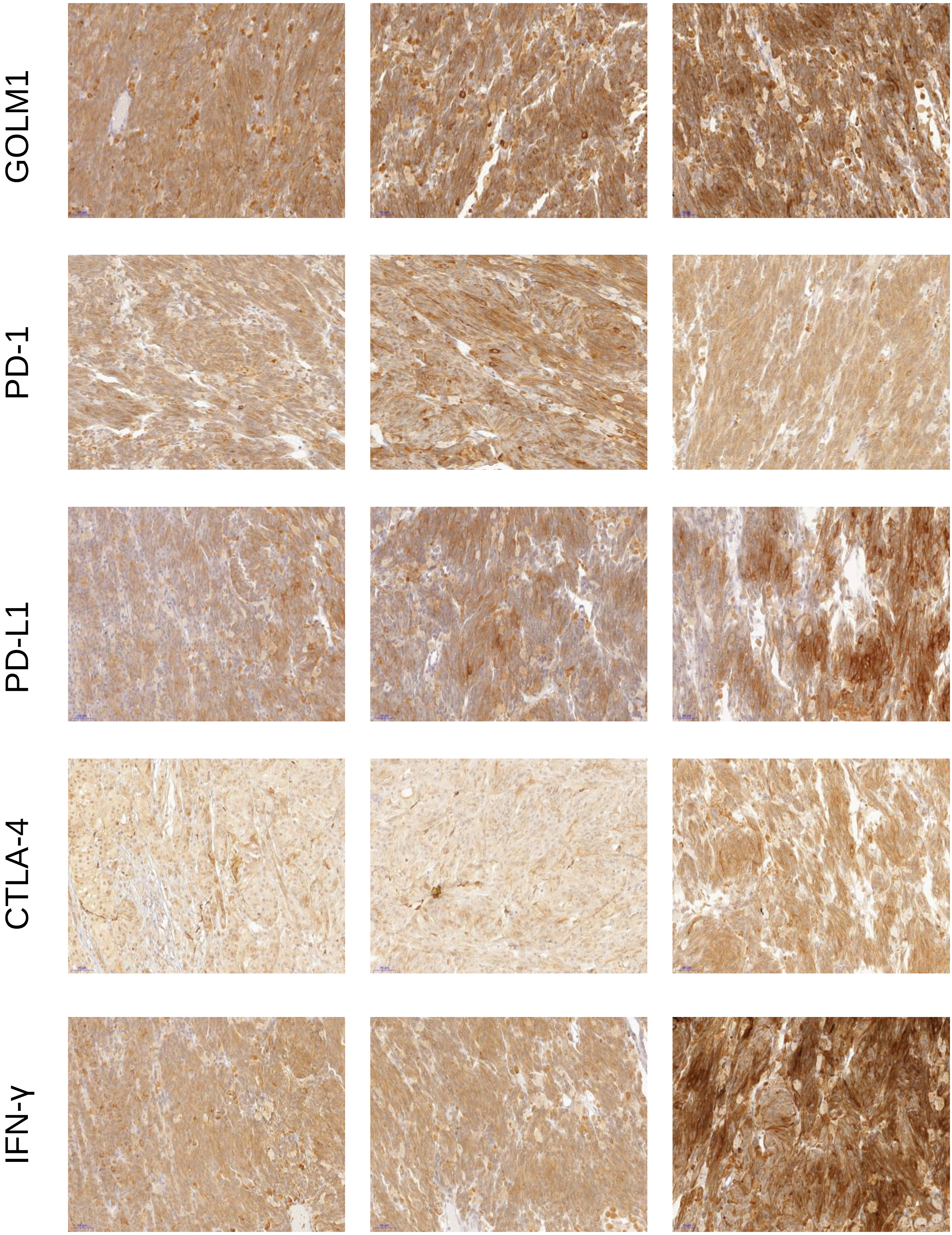

ID: 15

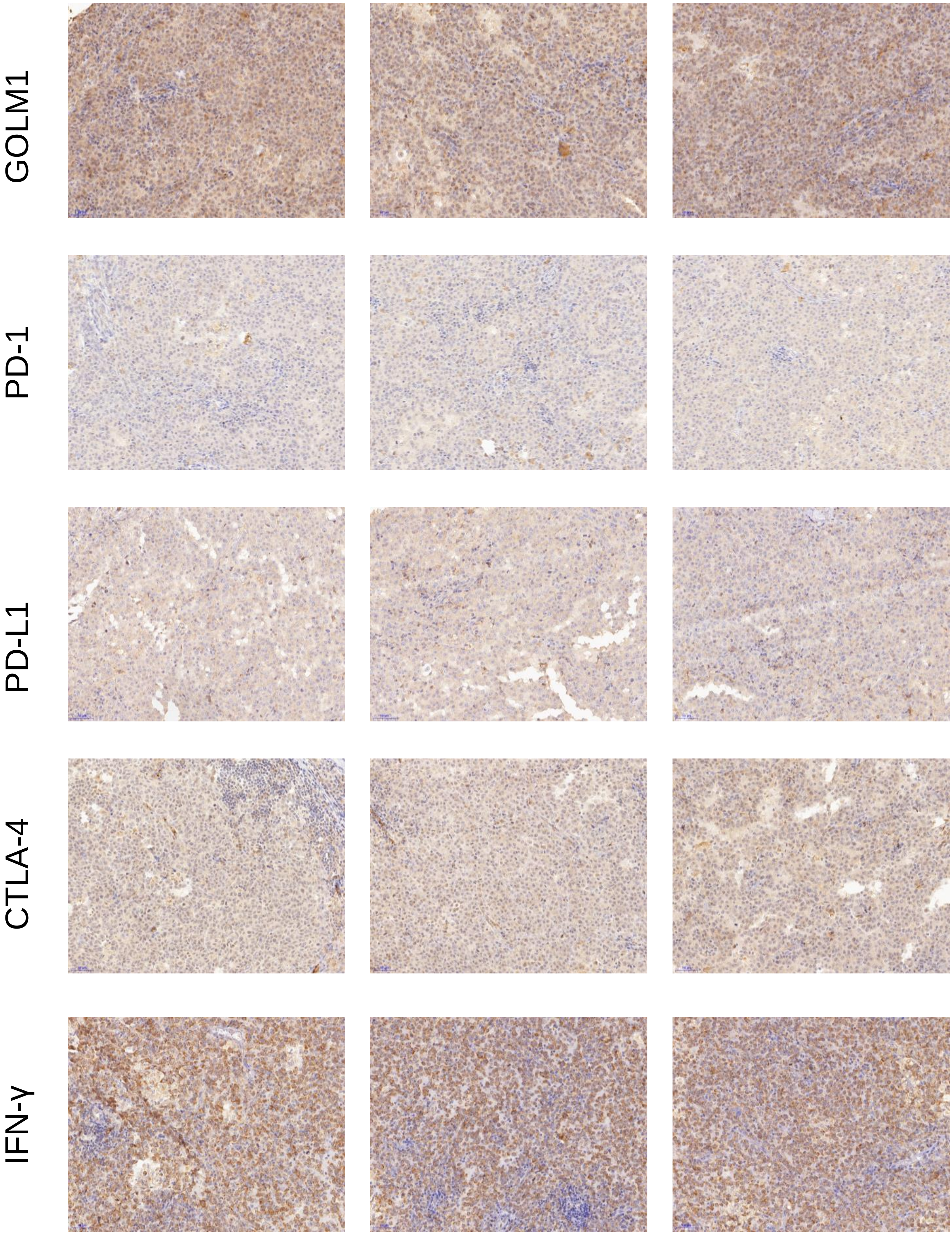

ID: 16

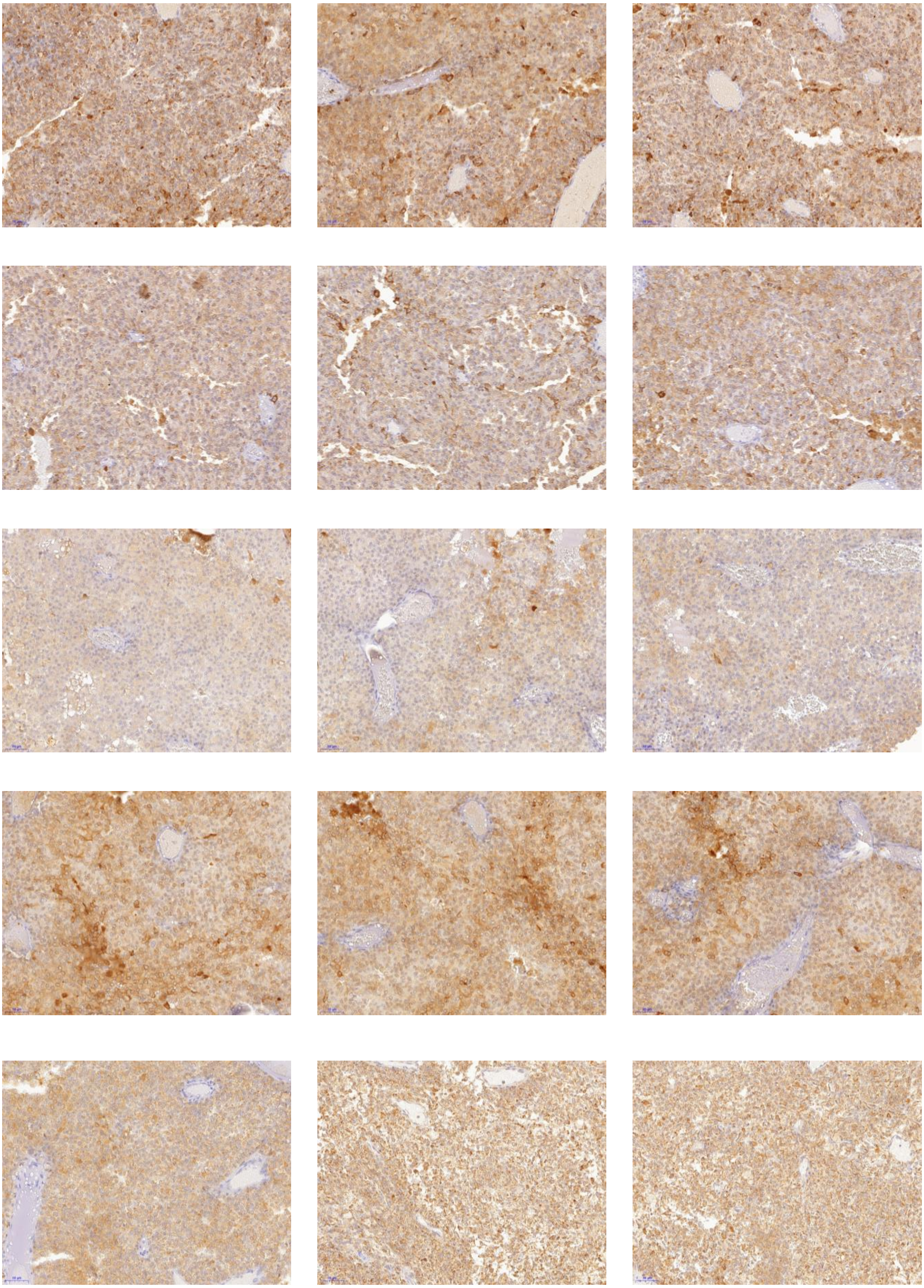

ID: 17

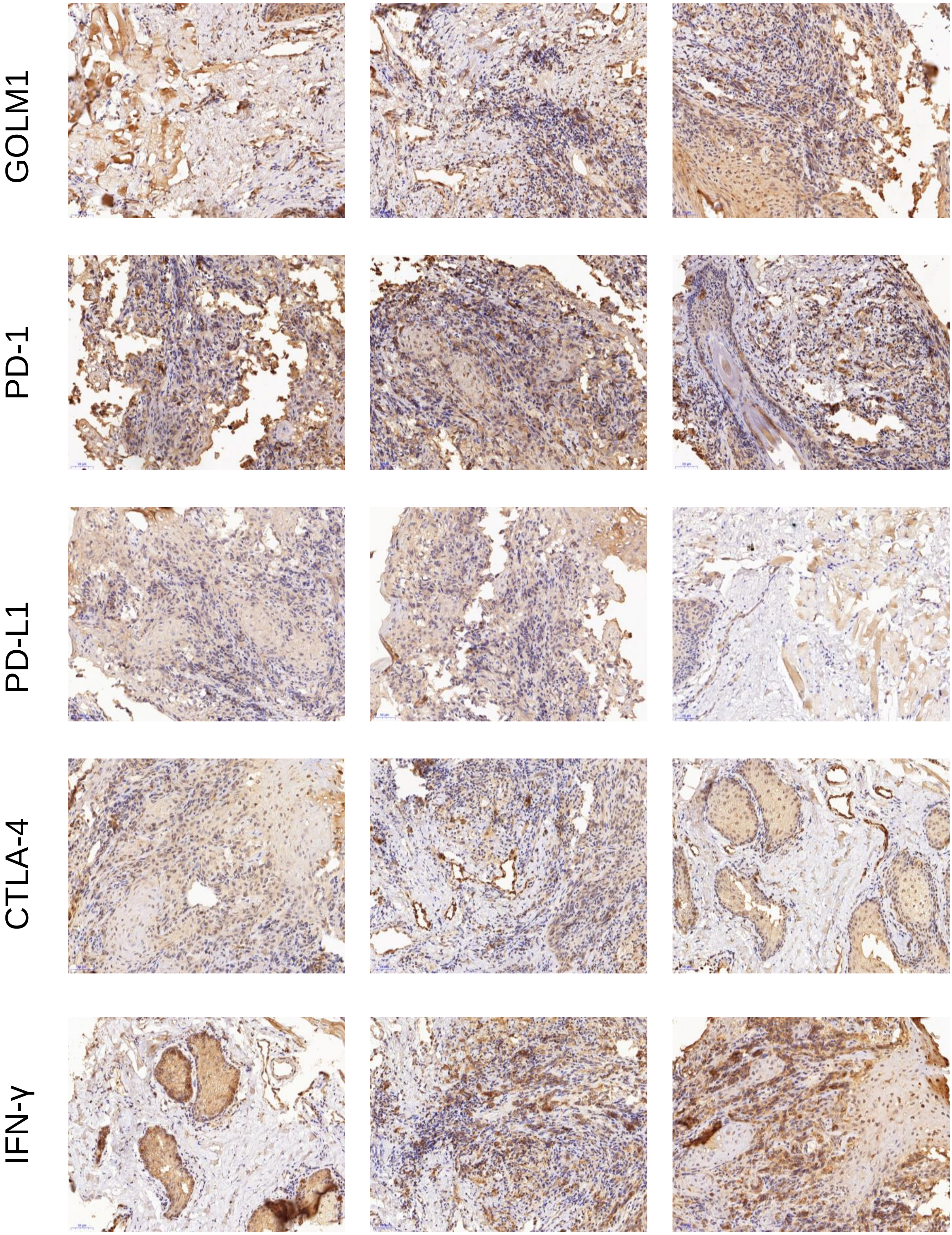

ID: 18

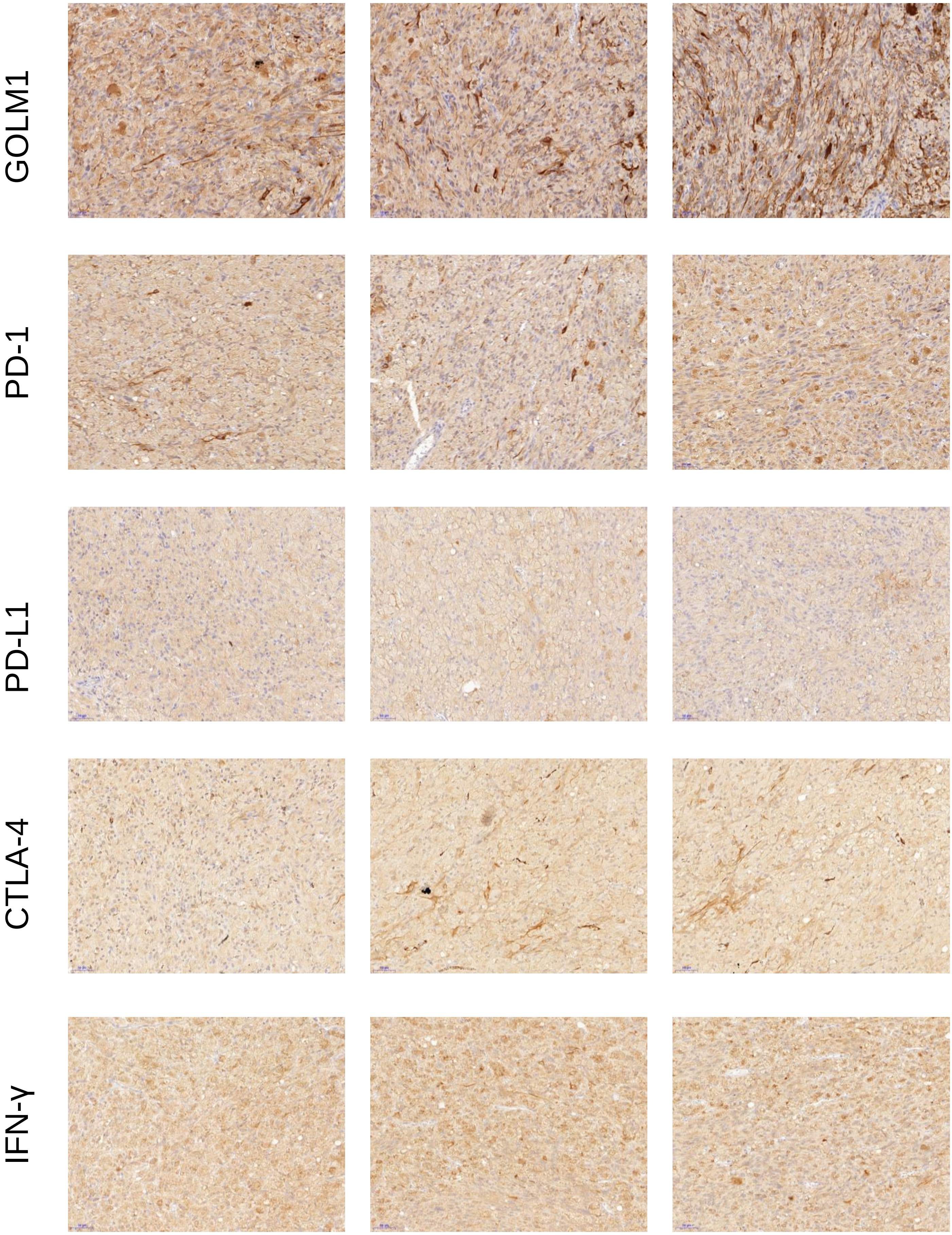

ID: 19

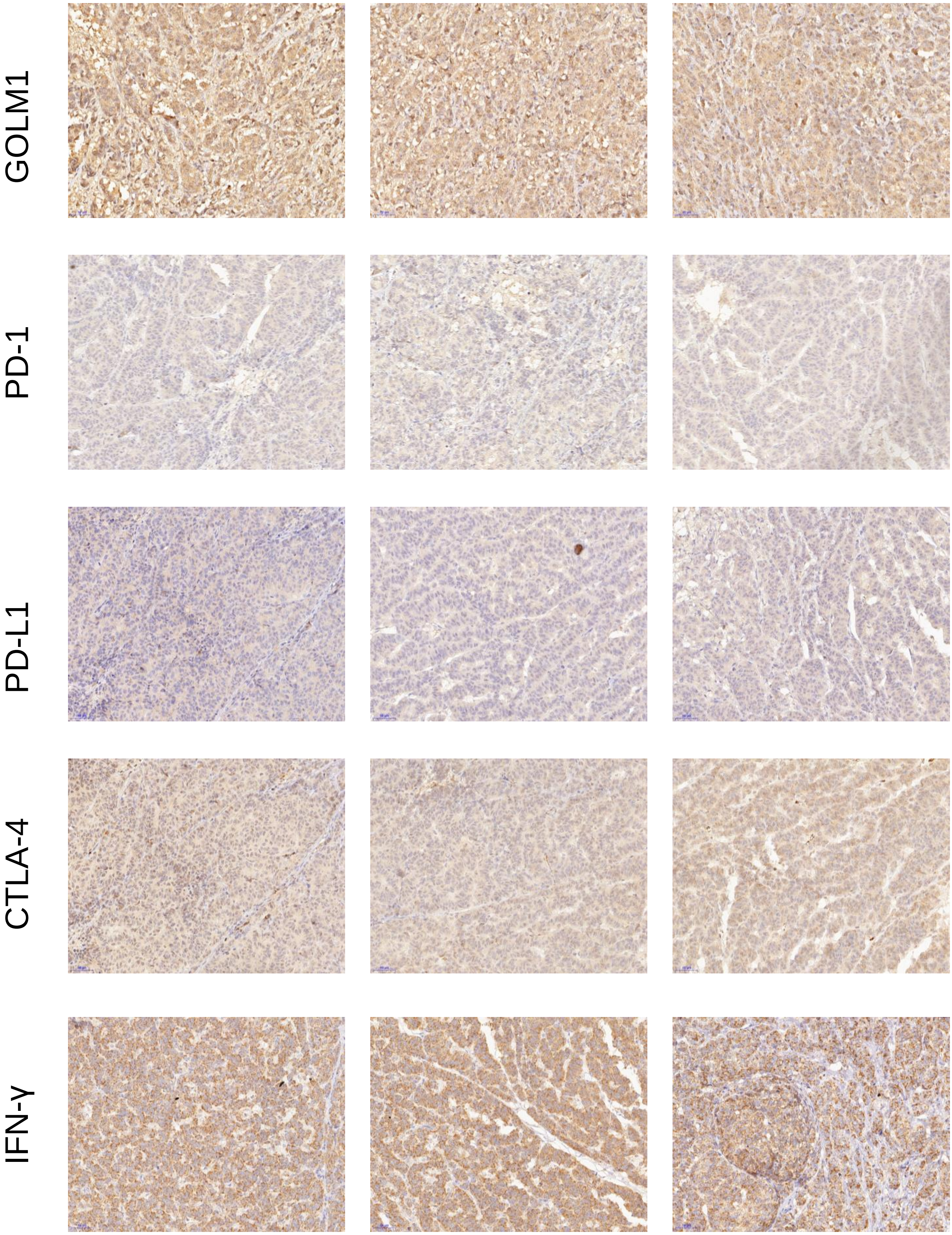

ID: 20

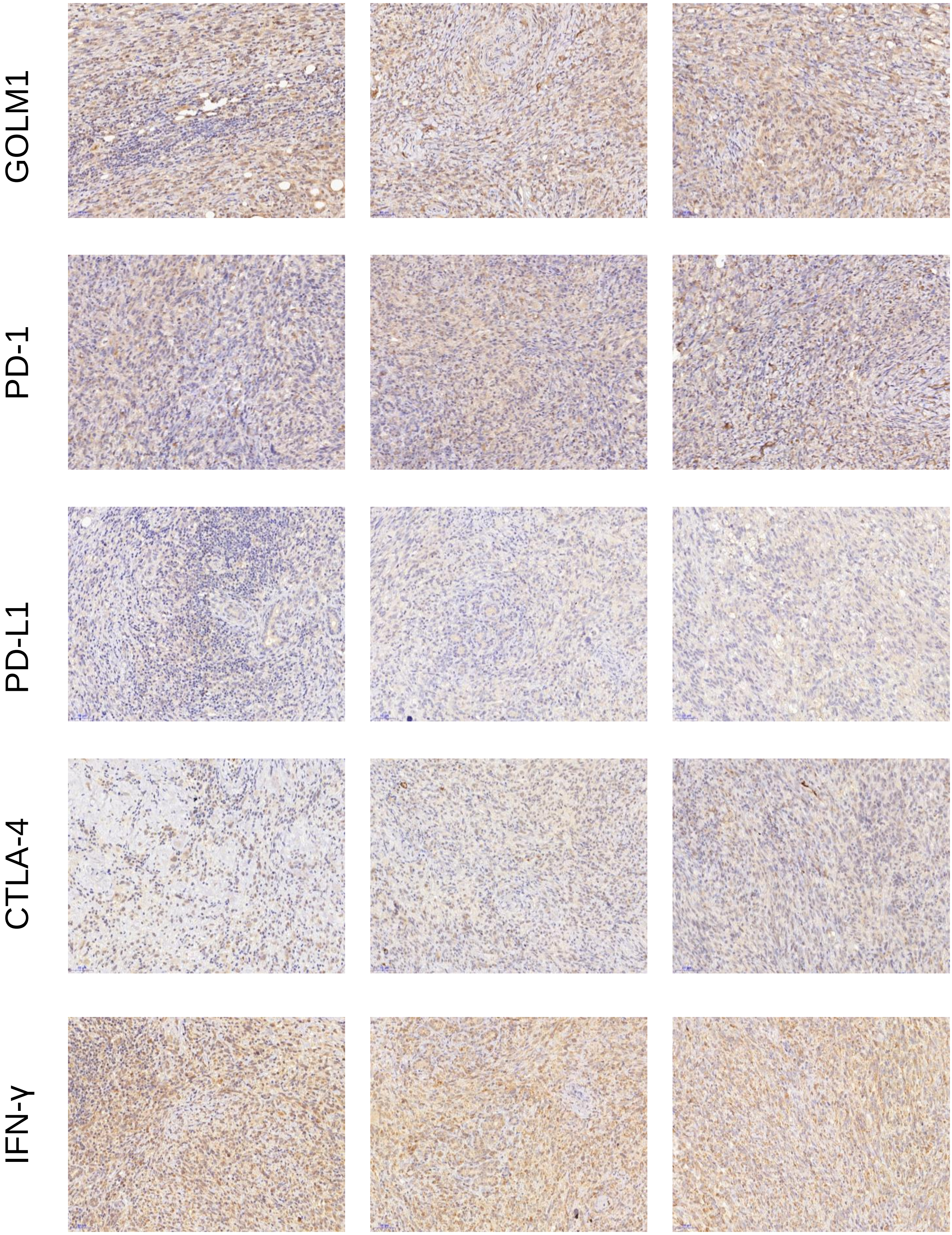

ID: 21

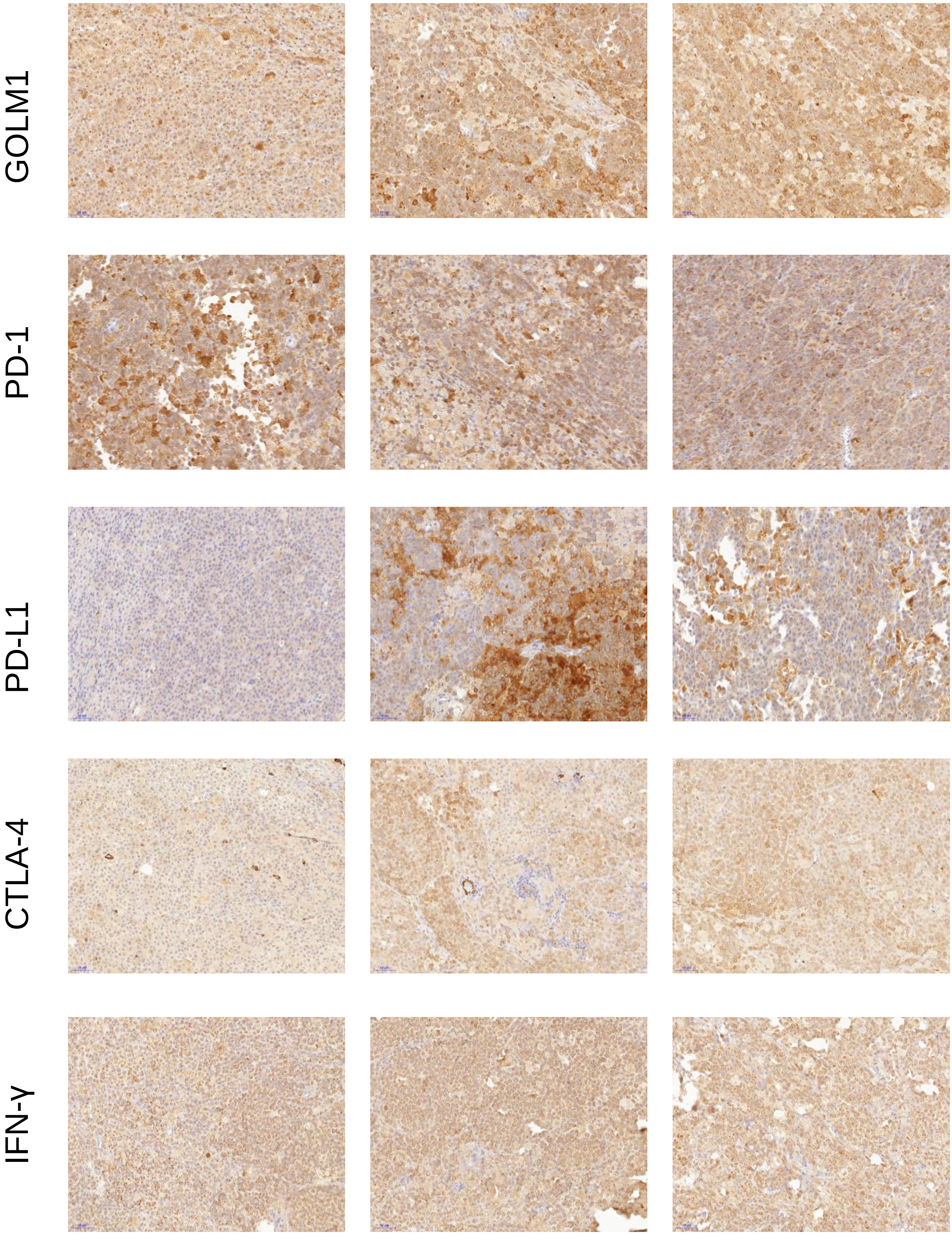

ID: 22

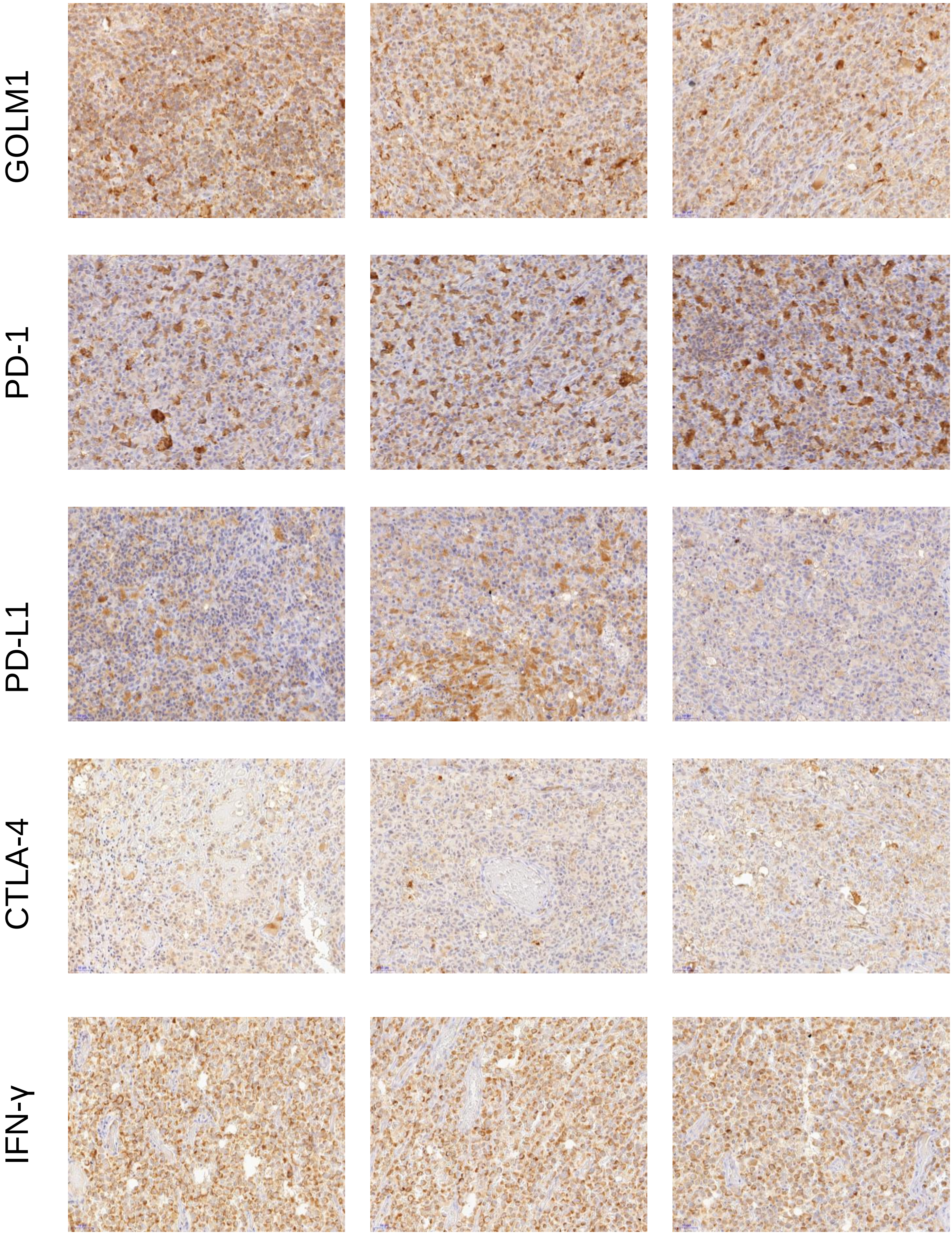

ID: 23

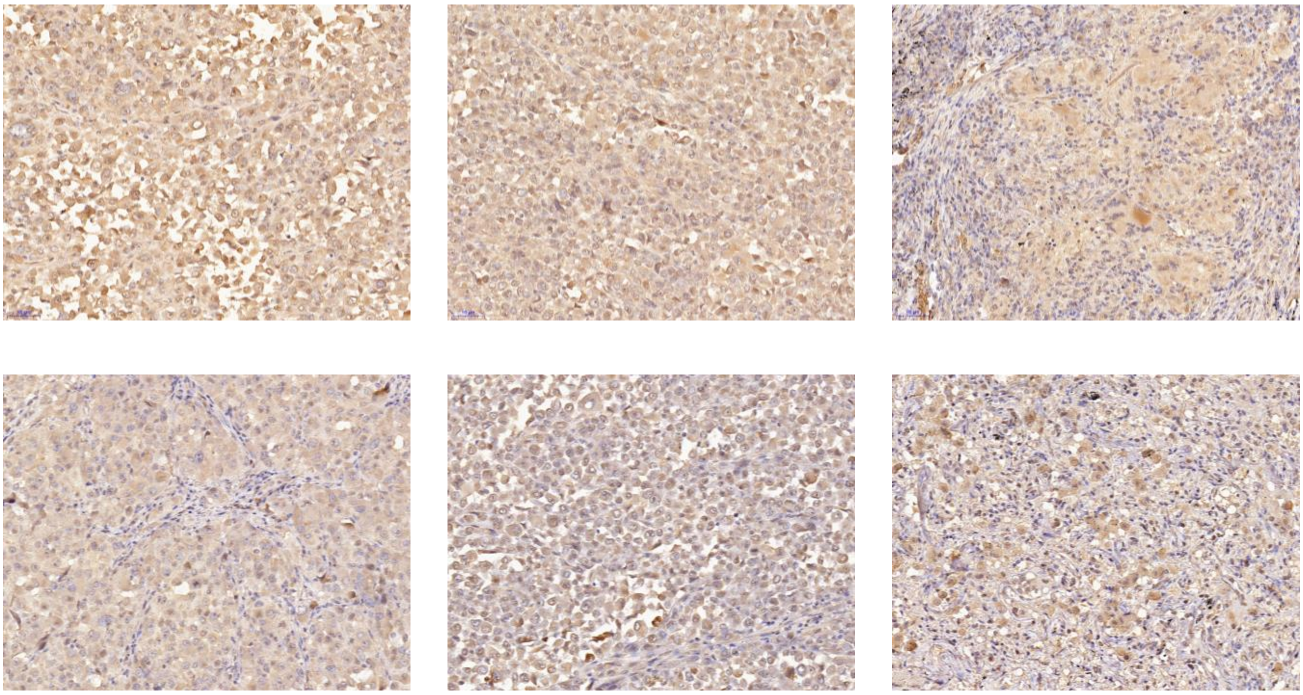

PD-L1

Patient 23 lacks enough slides for IHC, there for missing the results of PD-L1

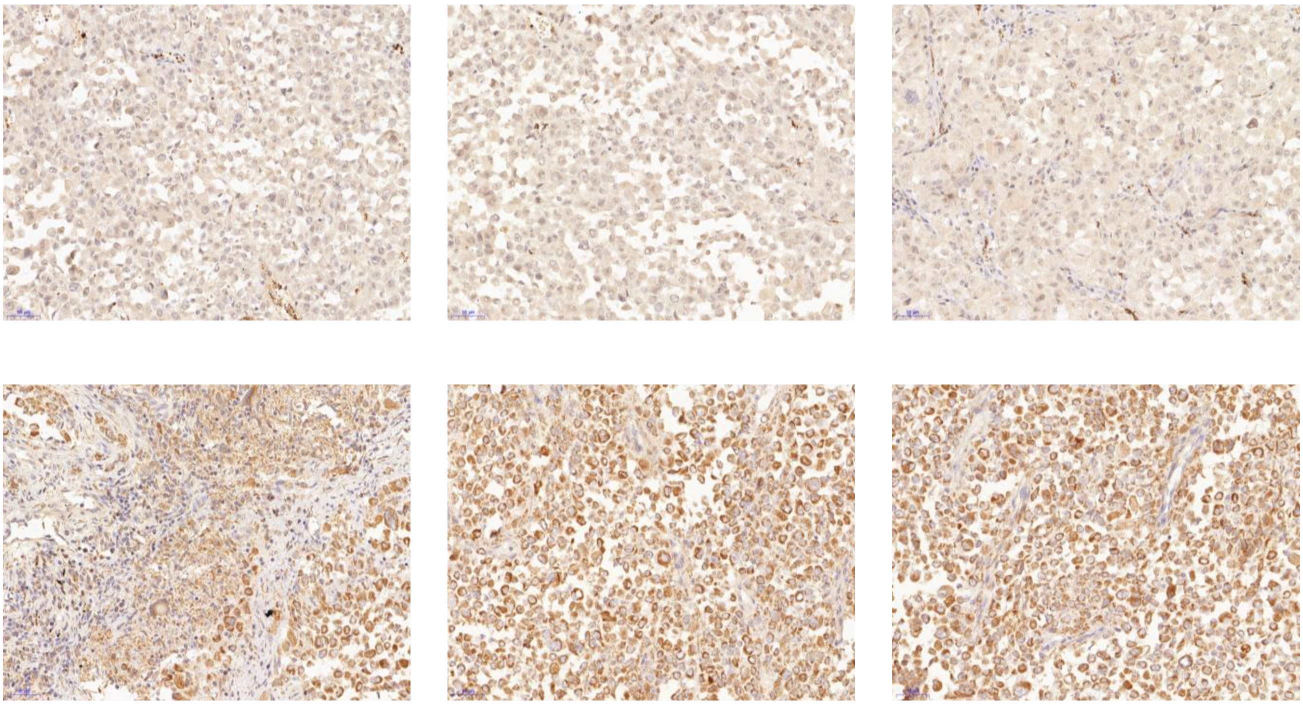

Supplement: Supplementary file 2 [file Presentation1.PDF]
